# Spontaneous functional network dynamics and associated structural substrates in the human brain

**DOI:** 10.3389/fnhum.2015.00478

**Published:** 2015-09-04

**Authors:** Xuhong Liao, Lin Yuan, Tengda Zhao, Zhengjia Dai, Ni Shu, Mingrui Xia, Yihong Yang, Alan Evans, Yong He

**Affiliations:** ^1^State Key Laboratory of Cognitive Neuroscience and Learning and IDG/McGovern Institute for Brain Research, Beijing Normal UniversityBeijing, China; ^2^Center for Cognition and Brain Disorders, Hangzhou Normal UniversityHangzhou, China; ^3^Neuroimaging Research Branch, National Institute on Drug Abuse, National Institutes of HealthBaltimore, MD, USA; ^4^McConnell Brain Imaging Centre, Montreal Neurological Institute, McGill UniversityMontreal, QC, Canada

**Keywords:** connectomics, functional dynamics, graph theory, hub, small-world, sliding window

## Abstract

Recent imaging connectomics studies have demonstrated that the spontaneous human brain functional networks derived from resting-state functional MRI (R-fMRI) include many non-trivial topological properties, such as highly efficient small-world architecture and densely connected hub regions. However, very little is known about dynamic functional connectivity (D-FC) patterns of spontaneous human brain networks during rest and about how these spontaneous brain dynamics are constrained by the underlying structural connectivity. Here, we combined sub-second multiband R-fMRI data with graph-theoretical approaches to comprehensively investigate the dynamic characteristics of the topological organization of human whole-brain functional networks, and then employed diffusion imaging data in the same participants to further explore the associated structural substrates. At the connection level, we found that human whole-brain D-FC patterns spontaneously fluctuated over time, while homotopic D-FC exhibited high connectivity strength and low temporal variability. At the network level, dynamic functional networks exhibited time-varying but evident small-world and assortativity architecture, with several regions (e.g., insula, sensorimotor cortex and medial prefrontal cortex) emerging as functionally persistent hubs (i.e., highly connected regions) while possessing large temporal variability in their degree centrality. Finally, the temporal characteristics (i.e., strength and variability) of the connectional and nodal properties of the dynamic brain networks were significantly associated with their structural counterparts. Collectively, we demonstrate the economical, efficient, and flexible characteristics of dynamic functional coordination in large-scale human brain networks during rest, and highlight their relationship with underlying structural connectivity, which deepens our understandings of spontaneous brain network dynamics in humans.

## Introduction

Over the past two decades, resting-state functional magnetic resonance imaging (R-fMRI), a promising functional imaging technique, has been widely used to non-invasively map the brain's intrinsic or spontaneous functional connectivity patterns (i.e., functional connectome) by measuring the correlations in blood oxygen level-dependent (BOLD) signals between regions during rest (Biswal et al., 1995; for reviews, see Fox and Raichle, 2007; Kelly et al., 2012). Recently, the combination of R-fMRI and graph-based network analyses has allowed us to quantitatively characterize the topological characteristics of functional networks in the human brain, such as small-worldness, modularity, and highly connected hub regions (for reviews, see Bullmore and Sporns, 2009; He and Evans, 2010; Bullmore and Sporns, 2012). These studies provide mechanistic insights into the functional organization principles of human brain networks in health and disease.

To date, the majority of R-fMRI studies on functional connectomics have focused mainly on static functional connectivity (S-FC) by computing inter-regional statistical associations in whole-scan time courses, implicitly neglecting the potential temporal fluctuations of functional interactions across time. Recently, emerging evidence suggests that functional coupling among regions is highly dynamic at a time scale of seconds to minutes (Chang and Glover, 2010; Kang et al., 2011; Kiviniemi et al., 2011; Hutchison et al., 2013b; Allen et al., 2014; Gonzalez-Castillo et al., 2014; Di and Biswal, 2015). The dynamic functional connectivity (D-FC) can capture the transition between different mental states (Allen et al., 2014) associated with changes in electroencephalograph (EEG) power (Tagliazucchi et al., 2012b; Chang et al., 2013), and provide novel insights into the pathophysiological mechanisms of neuropsychiatric disorders, such as Alzheimer's disease (Jones et al., 2012) and post-traumatic stress disorder (Li et al., 2014). Specifically, several recent R-fMRI studies have further demonstrated the dynamic characteristics of the topological organization of human brain networks such as fluctuating modular architecture (Jones et al., 2012; Allen et al., 2014) and sporadic intervals of high network efficiency (Zalesky et al., 2014; Di and Biswal, 2015). As mentioned above, the brain's functional networks include many non-trivial topological properties, such as small-worldness, which quantifies an optimal balance between information segregation and integration (Salvador et al., 2005; Bassett and Bullmore, 2006), and highly connected hubs, which play key roles in global brain communication (Achard et al., 2006; Buckner et al., 2009; Liao et al., 2013; van den Heuvel and Sporns, 2013). However, how these crucial topological properties of human brain networks vary spontaneously over time remains largely unknown.

A growing number of neuroimaging studies have suggested that the human brain's functional organization is sculpted by the underlying anatomical structure (see Reviews, Park and Friston, 2013; Wang et al., 2015). At the connection level, two regions often exhibit high temporal synchronization if they are directly linked by white matter tracts (Greicius et al., 2009; Honey et al., 2009; van den Heuvel et al., 2009). At the network level, the brain's functional and structural networks share topological organization, such as small-worldness and hubs (Bullmore and Sporns, 2009, 2012). Notably, all of these previous studies have been mainly confined to the exploration of the structural basis of static functional connectivity or networks. Since the anatomical structure constrains the propensity of inter-regional interactions, it's natural to expect that ongoing brain activities and the accompanying dynamic functional coordination may be shaped by the underlying structural organization (Deco et al., 2013). Until recently, Shen et al. (2015) have demonstrated the structural-dependence of the spontaneous dynamic functional coordination in macaques at both the local and global levels. However, for the human brain, very little is known about whether and how the dynamic connectivity patterns of functional networks are constrained by the underlying white matter tracts or structural connectivity (SC).

To address these issues, in the present study we employed BOLD R-fMRI data and graph theoretical approaches to systematically characterize the dynamic topological properties of human whole-brain functional networks. Further, we used diffusion tensor imaging (DTI) data to construct white matter structural networks in the same participants to reveal the structural substrates underlying these functional network dynamics. Specifically, to better track the brain network dynamics at a fine time scale, a multiband R-fMRI dataset with a sub-second sampling rate (TR = 645 ms) was used here, which provides additional temporal information regarding BOLD signal activities. Here, we sought to determine (i) how the topological organization of intrinsic or spontaneous functional brain networks changes over time at different levels (including connectional, global and nodal properties); and (ii) how the white matter structural connectivity underlies these dynamic network characteristics.

## Materials and methods

### Participants

The multiband R-fMRI and DTI data were selected from a publicly available dataset (http://fcon_1000.projects.nitrc.org/indi/pro/eNKI_RS_TRT/FrontPage.html) (Nooner et al., 2012). This dataset includes multi-modal imaging data of 24 participants (age: 34.4 ± 12.9, 6 females), which have been recently used in the test-retest reliability studies of functional homogeneity (Zuo et al., 2013), voxel-wise functional connectivity (Liao et al., 2013), and directed brain network analysis (Wu et al., 2013). In the current study, we discarded the data of 13 participants due to the potential effects of confounding health issues (current/historical psychiatric disorders, obvious brain atrophy or missing diagnostic information) or because of excessive head motion (see data preprocessing) (Table S1). Finally, the data of the remaining 11 healthy participants (age: 30.2 ± 9.6, 4 females) were used for subsequent network analyses (Table S1).

### Data acquisition

All of the participants underwent both R-fMRI and DTI scans twice, approximately 1 week apart, on a Siemens Trio 3.0 Tesla scanner, leading to repeated data in two sessions (i.e., Session 1 and Session 2). In each session, the R-fMRI scans were performed using three echo planar imaging protocols, each with different sampling rates (TR = 645, 1400, and 2500 ms), and participants were instructed to keep their eyes fixed on the cross on the screen. Here, we employed the multiband R-fMRI data with the sub-second sampling rate (TR = 645 ms), which provided finer temporal information regarding fluctuations in the BOLD signals. The detailed scanning parameters were as follows: time repetition [TR] = 645 ms; time echo [TE] = 30 ms; flip angle = 60°; 40 slices, multiband acceleration factor [MAF] = 4; field of view [FOV] = 222 × 222 mm^2^; voxel size = 3 × 3 × 3 mm^3^; total acquisition time = 9:46 min (i.e., 900 volumes). Because the last (900th) functional volume was missing in the R-fMRI data of four participants, we used the 899 volumes available for all participants. The parameters for the multiband DTI images were as follows: TR/TE = 2400/85 ms; flip angle = 90°; 64 slices, MAF = 4; FOV = 212 × 180 mm^2^; voxel size = 2 × 2 × 2 mm^3^; *b*-value = 1500 s/mm^2^, 128 gradient directions with 9 b = 0 images; total acquisition time = 5:58 min. Notably, DTI data of one participant was missing in the first session (Table S1). Additionally, a high-resolution T1-weighted image was also obtained for each participant with a magnetization prepared rapid gradient echo (MPRAGE) sequence: TR/TE = 2500/3.5 ms; flip angle = 8°; inversion time = 1200 ms; 192 slices; Matrix = 256 × 256; voxel size = 1 × 1 × 1 mm^3^. In the present study, the data from Session 1 were used for the main analyses, and the data from Session 2 were used for the validation analyses.

### Data preprocessing and analysis

#### Multiband R-fMRI data

Data preprocessing was performed using Statistical Parametric Mapping (SPM8, http://www.fil.ion.ucl.ac.uk/spm) and Data Processing Assistant for Resting-State fMRI (DPARSF) (Yan and Zang, 2010). First, 16 volumes in the first 10 s were discarded for signal equilibrium and to allow the participants' adaption to the scanning environment. The remaining data were corrected for head motion and participants with large head motion (2 mm or > 2°) were excluded (Table S1). Subsequently, the motion-corrected images were spatially normalized to Montreal Neurological Institute (MNI) space using an optimum, 12-parameter affine transformation and nonlinear deformations (Ashburner and Friston, 1999) and then resampled to 3-mm isotropic voxels. Linear trends were further removed from the normalized images, and then the images were temporally band-pass filtered (0.01–0.1 Hz). Finally, several confounding factors were regressed out as covariates using multiple linear regression, including 24 head-motion parameters (Friston et al., 1996) and cerebrospinal fluid (CSF), white matter (WM) and global brain (Fox et al., 2005) signals. The residual time series were used for further network analysis.

#### Multiband DTI data

The preprocessing of the DTI data consisted of eddy current and motion artifact correction, estimation of the diffusion tensor, calculation of fractional anisotropy (FA) and diffusion tensor tractography. The first three steps were performed with the FDT toolbox in FSL (http://www.fmrib.ox.ac.uk/fsl) (Smith et al., 2004) as follows. First, an affine transformation was applied to align each diffusion-weighted image to the *b* = 0 image to correct for eddy current distortions and motion artifacts. Second, the diffusion tensor was calculated by solving the Stejskal and Tanner equation (Basser et al., 1994), and the reconstructed tensor matrix was diagonalized to obtain three eigenvalues (λ1, λ2, and λ3) and corresponding eigenvectors. Finally, the FA value was calculated voxel by voxel using the three eigenvalues of the tensor matrix (Basser and Pierpaoli, 1996). Subsequently, the structural T1-weighted image was segmented into gray matter (GM), WM and CSF in the CIVET pipeline (http://mcin-cnim.ca/neuroimagingtechnologies/civet/) and registered to the *b* = 0 image to obtain the white matter mask in DTI native space. Diffusion tensor tractography was then implemented in Diffusion Toolkit (http://trackvis.org/) using the “fiber assignment by continuous tracking (FACT)” method (Mori et al., 1999) by seeding each voxel of the white matter mask. Specifically, given a voxel, eight seeds were placed evenly within the volume of the voxel (van den Heuvel and Sporns, 2011). Then each fiber was reconstructed by tracking from each seed following the main diffusion direction of the current voxel into the next, thus reconstruction was terminated if the fiber turned with an angle >45° or went out of the white matter mask (Mori et al., 1999). After tracking from all white matter voxels of the brain, a large member of fiber bundles was reconstructed and formed the whole-brain WM fibers for each participant.

#### Regional parcellations

To derive inter-regional functional and structural connectivity, we parcellated the cerebral cortex into different regions of interest (ROIs). Given that the topological organization of the brain networks could be affected by different spatial parcellations (Wang et al., 2009; Fornito et al., 2010; Zalesky et al., 2010), in the present study we investigated the functional and structural connectomics using two different regional parcellation schemes representing low and high spatial resolution, respectively. In the first parcellation scheme, 90 ROIs were obtained based on the automated anatomical labeling atlas (AAL-90; for details of the ROIs, see Table S2) (Tzourio-Mazoyer et al., 2002); in the second parcellation scheme, 1024 ROIs with uniform sizes were obtained based on a random parcellation (H-1024) (Zalesky et al., 2010). For each parcellation scheme, we investigated the dynamic functional connectivity (D-FC) and structural connectivity (SC) among regions. Notably, the analysis of the H-1024 brain networks was identical to that of the AAL-90 brain networks.

### Dynamic functional connectivity analysis

#### Extraction of D-FC

**Figure 1:**
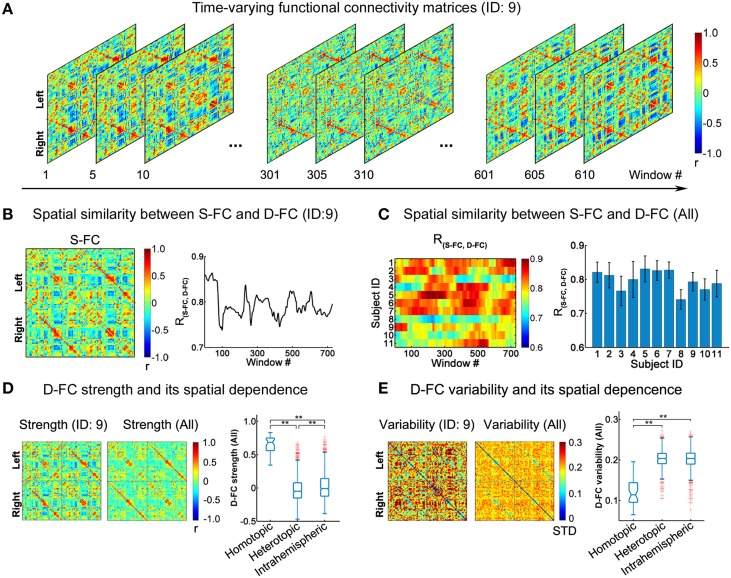
**Spatial patterns of D-FC matrices over time. (A)** Transient D-FC matrices at different sliding windows for one representative participant (ID: 9). Regions of interest (ROIs) were indexed in an order of left (ROIs: 1-45) to right (ROIs: 46-90) hemisphere (for details of the ROIs, see Table S2). **(B)** Static functional connectivity (S-FC) matrix of the same participant (ID: 9) (left) and its spatial similarity with the D-FC matrices over time (right). *R*_(*S*−*FC, D*−*FC*)_ denotes the spatial similarity between the S-FC and the D-FC matrices obtained using Pearson's correlation analysis following Fisher's r-to-z transformation. **(C)** Time-varying spatial similarity between the S-FC and the D-FC matrices for all participants (left) and associated temporal mean and temporal variability across windows (right). Error bars represent the standard deviation across windows. **(D)** D-FC strength and its spatial dependence for one participant (ID: 9) and for all participants. **(E)** D-FC variability and its spatial dependence for one participant (ID: 9) and for all participants. In **(D, E)**, the D-FC strength/variability matrix for all participants was generated by averaging the individual strength/variability matrices across participants. The statistical difference between any two categories of D-FC was tested using permutation tests (^**^Bonferroni corrected *p* < 0.001, 10,000 permutations). S-FC, static functional connectivity; D-FC, dynamic functional connectivity.

For each participant, we employed a commonly used sliding window approach to estimate the D-FC of every pair of ROIs (Kiviniemi et al., 2011; Jones et al., 2012; Tagliazucchi et al., 2012b; Hutchison et al., 2013b; Allen et al., 2014; Zalesky et al., 2014). Briefly, the time course of each ROI was first obtained by averaging the residual time courses of all voxels within the ROI. Then, a sliding rectangular window with a fixed length was selected, and each ROI's time course within this window was used to estimate the D-FC of interest. Within a given sliding window *t*, a symmetric *N* × *N* D-FC matrix, *R*_*t*_ = [*r*_*t*_(*i,j*)], was generated, where *N* denotes the number of ROIs considered (*N* = 90 for the AAL-90 network and *N* = 1024 for the H-1024 network), and *r*_*t*_(*i,j*) represents the Pearson's correlation coefficient between the time courses of two ROIs, *i* and *j*. Here, we utilized a sliding window with a length of 155 TRs (i.e., 100 s), which allows us to estimate D-FC over the low-frequency band of interest (0.01–0.1 Hz) with an adequate number of time points (at least one period), and simultaneously to capture the time-varying information of the D-FC. This window was shifted in time with a step size of one TR (i.e., 645 ms), resulting in 729 D-FC matrices (*R*_*t*,_ *t* = 1, 2, …, 729) for each participant (Figure 1; Video S1). We also assessed the effects of different sliding window lengths on the main results (see “Validation analysis”).

#### Temporal characteristics of D-FC

We studied the time-varying features of the D-FC matrices as follows: (i) To estimate the temporal fluctuations of the whole-brain D-FC patterns, for each participant, we constructed the *N* × *N* static functional connectivity (S-FC) matrix as a reference, which was represented by Pearson's correlation coefficients over the whole-scan time courses. The time-varying spatial similarity between the S-FC and D-FC matrices, *R*_(*S*−*FC, D*−*FC*)_, was calculated via Pearson's correlation analysis within each sliding window. Notably, prior to the correlation analysis, Fisher's r-to-z transformation (Zar, 1996) was performed on all entries in both matrices to improve the normality of the correlation distribution. (ii) To quantitatively ascertain the temporal features of the D-FC between every pair of ROIs (*i* and *j*), two measures, temporal mean (tMean) and temporal variability (tVar), were estimated as follows:

(1) $$\begin{array}{r} tMean_{\text{D}-\text{FC}\left(\text{i},\text{j}\right)}=\frac{1}{T}\overset{T}{\underset{t=1}{\sum }}r_{t}\left(i,j\right), \end{array}$$

(2) $$\begin{array}{r} tVar_{\text{D}-\text{FC}\left(\text{i},\text{j}\right)}=\sqrt{\frac{1}{T-1}\overset{T}{\underset{t=1}{\sum }}{\left(r_{t}\left(i,j\right)-tMean_{\text{D}-\text{FC}\left(\text{i},\text{j}\right)}\right)}^{2}}, \end{array}$$

where, *r*_*t*_(*i,j*)represents the D-FC strength between ROIs *i* and *j* within a given window *t*, and *T* denotes the total number of sliding windows (here, *T* = 729) for each participant. Therefore, for each participant an *N* × *N* D-FC strength matrix and an *N* × *N* D-FC variability matrix were generated. (iii) To further explore the spatial dependence of D-FC strength and variability, we generated the average D-FC strength and variability matrices across individuals and then classified all of the D-FCs into three broad categories based on the inter-regional spatial relationships (Stark et al., 2008): homotopic edges, representing the connections between homologous brain areas in two hemispheres; heterotopic edges, representing the connections between non-homologous brain areas in two hemispheres; and intrahemispheric edges, representing the connections between brain areas within the same hemisphere. Next, differences in D-FC strength or variability between any two edge categories were tested using permutation tests, during which the D-FC group assignments were randomly shuffled 10,000 times to obtain the null distribution. The Bonferroni approach was applied to correct for multiple testing across all of the category pairs examined (three in total).

### Topological analyses of dynamic functional brain networks

#### Construction of dynamic functional networks

To assess the time-varying topological properties of whole-brain functional networks, for each participant, we first generated a set of binary D-FC matrices (i.e., binary functional networks) over time through a thresholding approach and then performed graph-based network analysis. Specifically, for a given sliding window *t*, any correlation *r*_*t*_(*i*,*j*) greater than a given threshold τ was retained as an edge connecting ROIs *i* and *j*; no edge existed otherwise. The correlation threshold τ was determined via a Bonferroni correction with *p*_*corr*_ < 0.01, which preserved the significantly existing correlations, producing dynamic functional networks with different edge numbers. Moreover, the edges with negative correlations were ignored during the network topology analysis, as their physiological meaning is not clear yet (Fox et al., 2009; Murphy et al., 2009; Weissenbacher et al., 2009; Adachi et al., 2012). We also assessed the effect of different thresholding strategies on the main results (see “Validation analysis”).

#### Network topology analysis

All network analyses were performed using the in-house Gretna package (http://www.nitrc.org/projects/gretna). For the D-FC network within each sliding window, we calculated both global and nodal network metrics (for review, see Rubinov and Sporns, 2010), to characterize their overall architecture and nodal centrality, respectively. (i) The global network metrics included connectivity density or sparsity (*S*), small-world attributes (clustering coefficient, *C*_*p*_, and characteristic path length, *L*_*p*_), assortativity (α), and their normalized versions using random networks (γ, λ and α_*z*−*score*_). Briefly, the density or sparsity (*S*) of a network characterizes the number of existing edges, indicating the total wiring costs of the network. The clustering coefficient of a node reflects the fraction of triangles around this node, indicating the extent of local information segregation (Watts and Strogatz, 1998). Hence the clustering coefficient (*C*_*p*_)of a network characterizes the prevalence of local clustering in the network, reflecting the overall capability for information segregation. The characteristic path length (*L*_*p*_) describes the average length of the shortest paths linking any two nodes in the network, measuring the capability for information integration. Typically, a small-world network architecture supports the balance between information segregation and integration with a low wiring cost, and is characterized by high local clustering (γ = *C*_*p*_*/*<*C*${}_{p}^{rand}$>>>1) and short path length (λ = *L*_*p*_/ < *L*${}_{p}^{rand}$> ~ 1), leading to a small-world index σ = γ/λ > 1 (Humphries and Gurney, 2008). Specifically, < *C*${}_{p}^{rand}$ > and < *L*${}_{p}^{rand}$ > were the average of *C*_*p*_ and *L*_*p*_, respectively, estimated from 100 matched random networks conserving the same number of nodes, edges and the same degree distribution as the real brain networks (Maslov and Sneppen, 2002). Assortativity (α) describes the similarity of degree values for any two connected nodes (Newman, 2002). Positive values of α indicate nodes possessing large degrees prefer to connect with each other to form a relatively resilient core, and vice versa. Assortativity is considered significant if the normalized value α_*z*−*score*_ = (α^*real*^− < α^*rand*^ >)/*std*(α^*rand*^) > 1.64 (i.e., one-tailed *t*-test with *p* < 0.05), where < α^*rand*^ > and *std*(α^*rand*^) denote the mean and the standard deviation of the α values, respectively, estimated from 100 matched random networks. (ii) For regional topological properties, we employed nodal degree centrality due to its higher test-retest reliability in the functional networks than other nodal metrics (Wang et al., 2011). The degree *k*_*i*_ of node *i* is defined as the number of edges directly connected to this node, indicating its role in the information communication in the network.

#### Temporal characteristics of network topology

The temporal characteristics of each network metric were estimated across windows within each participant, which were further averaged across individuals for group-level analysis. Briefly, for each global metric, including *S, C*_*p*_, *L*_*p*_, γ, λ, σ, and α, we estimated a normalized histogram across windows. For nodal degree, we identified the hub regions for each sliding window, the degree values of which exceeded the mean value across the brain. Then, we counted the occurrence probability (i.e., ratio of sliding windows) as functional hubs for each region, and identified functionally persistent hub regions that possessed occurrence probability >50% over time. Finally, we estimated the temporal mean (*tMean*) and temporal variability (*tVar*) of each global or nodal metric over time as follows:

(3) $$\begin{array}{r} tMean_{NET}=\frac{1}{T}\overset{T}{\underset{t=1}{\sum }}NET_{t}, \end{array}$$

(4) $$\begin{array}{r} tVar_{NET}=\sqrt{\frac{1}{T-1}\overset{T}{\underset{t=1}{\sum }}{\left(NET_{t}-tMean_{NET}\right)}^{2}}, \end{array}$$

where, *NET* represents the global or nodal metric of interest, *NET*_*t*_ denotes its value for a given sliding window *t*, and *T* denotes the total number of sliding windows (here, *T* = 729) for each participant.

### Relationship between structural and dynamic brain networks

Recent studies have suggested that the intrinsic or spontaneous functional organization of the brain is shaped by the underlying structural architecture (Hagmann et al., 2008; Honey et al., 2009; See Reviews Deco et al., 2011, 2013; Park and Friston, 2013; Wang et al., 2015). Here, we reconstructed human white matter structural networks using DTI data from the same participants, and then investigated the relationship between dynamic functional networks and structural networks at different levels (including connectional, global and nodal properties).

#### Construction of structural networks

The network construction approaches were the same as our previous procedures (Gong et al., 2009; Shu et al., 2011). Briefly, the nodes were first obtained using SPM8 (http://www.fil.ion.ucl.ac.uk/spm) based on the parcellation schemes mentioned above (AAL-90 or H-1024). Specifically, the registered T1 image was nonlinearly transformed into the ICBM152 T1 template in the MNI space, and the corresponding transformation matrix was inversed to warp the parcellation scheme (AAL-90 or H-1024) from the MNI space to the DTI native space using a nearest-neighbor interpolation method to preserve discrete labeling values. Second, any two ROIs in DTI native space were considered structurally connected if at least one fiber streamline existed with two end-points locating in these two regions, respectively (Shu et al., 2011; Zalesky et al., 2011). Hence, for each participant, an undirected weighted SC matrix was constructed, indicating the presence or absence of SC between region pairs, as well as the SC strength. Specifically, for each SC its strength was denoted by the normalized number of streamlines, which was calculated by dividing the number of interconnecting streamlines by the mean volume of the two connected regions. Additionally, to balance the inter-individual variance in structural connections, we identified significantly consistent connections across participants by performing a nonparametric one-tailed sign test on the normalized streamline number (Gong et al., 2009). For each pair of ROIs, the sign test was performed with the null hypothesis that no connection existed (*p* < 0.05). Non-zero connections were preserved and assigned the average SC strength across participants to generate the group-level SC network (i.e., backbone).

#### Relationship between D-FC and SC networks

We explored the relationship between D-FC and SC networks at the following three levels. (i) Connection level. The relationship between D-FC and SC was performed at both individual and group levels. Specifically, for each participant, we examined whether there was a significant difference in D-FC strength or variability between region pairs with direct SC and those without direct SC. Here, a non-parametric permutation test was employed, where D-FC group assignments were randomly shuffled 10,000 times between the two categories of SC present and SC absent. Then, for the region pairs with direct SC, the relationship between SC strength and D-FC strength or variability was estimated using Pearson's correlation analysis. Prior to correlation analysis, a Gaussian resampling method was separately applied to all of the measures to improve the normality of their distributions. Similar analysis was also performed at the group level, based on the SC backbone network. (ii) Global-topology level. For each participant, we first estimated the global network metrics, including *C*_*p*_, *L*_*p*_, γ, λ, σ, and α, for the binary structural networks. Then, for each global metric, we used an across-subject Pearson's correlation analysis to examine the relationship between the SC networks and the temporal features of the D-FC networks, including temporal mean and temporal variability. (iii) Nodal-topology level. For each participant, we calculated nodal degree centrality in the binary structural network and identified the structural hub regions whose degree values exceeded the mean value across the brain. Then, we counted the occurrence probabilities of structural hubs across individuals, and identified structurally consistent hub regions with occurrence probabilities >50%. Furthermore, for all participants, we explored the potential constraints of both structural hub probability and structural degree centrality on the temporal variability of functional degree centrality via across-node correlation analysis. Because the values of structural hub probability were discrete, Spearman's correlation analysis was performed in these correlation analyses. Notably, during steps (ii) and (iii), network metrics were estimated for binary structural networks ignoring the SC strength, to ensure the comparability of network topology between structural and dynamic functional networks.

### Validation analysis

We evaluated whether the main findings were affected by scanning session or by different analysis strategies (e.g., the window length, the correlation thresholding strategy for network construction, and the network type). Given that the main results were compatible for low-resolution (AAL-90) and high-resolution (H-1024) brain networks (see Results), the validation analyses were performed on the low-resolution AAL-90 networks to reduce the computational burden. The relevant procedures are described as follows: (i) Scanning session. To validate our main findings, we performed the same network analysis on the multiband R-fMRI and DTI dataset in Session 2 of the same participants, which was scanned approximately 1 week after Session 1. (ii) Window length. In the current study, a commonly used sliding window approach was employed to capture the dynamics of D-FC (Kiviniemi et al., 2011; Jones et al., 2012; Tagliazucchi et al., 2012b; Hutchison et al., 2013b; Allen et al., 2014; Zalesky et al., 2014). However, so far, the choice of window length remains controversial (Hutchison et al., 2013a); various window lengths have been used in the previous studies (e.g., 30 ~240 s in Hutchison et al., 2013b). In the main analyses, we employed a sliding window with a length of 100 s, to capture the primary low-frequency BOLD signal fluctuations. Moreover, two additional window lengths (50–150 s) were considered to validate the main results. (iii) Correlation thresholding strategy. During functional network construction, various strategies can be used to threshold the functional correlation matrix to derive a connectivity matrix representing the functional network (Wang et al., 2010). Here, we employed a fixed correlation threshold for all of the windows, which was determined by a significance criterion—Bonferroni correction with *p*_*corr*_ < 0.01. To assess the effect of different correction thresholds, we conducted analyses at two additional thresholds of significance *p*_*corr*_ < 0.05 and 0.001. Moreover, we also generated the D-FC brain networks using different connectivity densities or sparsities (10, 15, and 20%), to ensure the same number of connections in the D-FC networks across all of the windows. (iv) Network type. In the main analyses, the network analyses were performed on binary brain networks, ignoring the differences in edge weights or connection strength. To assess whether our main results depended on the edge weights, we performed the same graph-based analyses on weighted D-FC networks and structural networks, where the weighted D-FC networks were obtained using the Bonferroni correction approach (*p*_*corr*_ < 0.01), but retaining the correlation values for the significant connections.

## Results

In the current study, we constructed dynamic functional networks and structural networks using both low-resolution (AAL-90) and high-resolution (H-1024) parcellations, and further investigated the dynamic characteristics of the functional networks and their associations with structural connectivity features. Given that most of the findings obtained from the AAL-90 and H-1024 brain networks were compatible, we mainly reported the results from the AAL-90 brain network analyses.

### Dynamic functional connectivity among regions

Using the sliding window approach, we generated individual inter-regional D-FC matrices, fluctuating at a sub-second time scale, for the whole brain. Figure 1A illustrates the transient D-FC matrices of several windows (i.e., time) for one representative participant (ID: 9; see Video S1 for all of the D-FC matrices across time). Notably, although the D-FC matrices varied across windows, they maintained high spatial similarity [*R*_(*S*−*FC, D*−*FC*)_ > 0.7] with the S-FC matrix (Figure 1B). Similar features were also observed for other participants (Figure 1C), with the mean *R*_(*S*−*FC, D*−*FC*)_ across windows exceeding 0.7, suggesting the existence of intrinsic rules (e.g., potential structural constraints) underlying the dynamic functional coordination. To further ascertain the temporally fluctuating characteristics of these D-FC matrices, for each participant, we computed the connectivity strength and temporal variability of each D-FC and obtained the D-FC strength and variability matrices, respectively (Figures 1D,E). At the group level, we found that homotopic D-FC showed significantly greater connectivity strength and smaller temporal variability than either heterotopic or intrahemispheric D-FC (both Bonferroni-corrected *p*s < 0.001, 10,000 permutations).

### Dynamic global properties of functional brain networks

**Figure 2:**
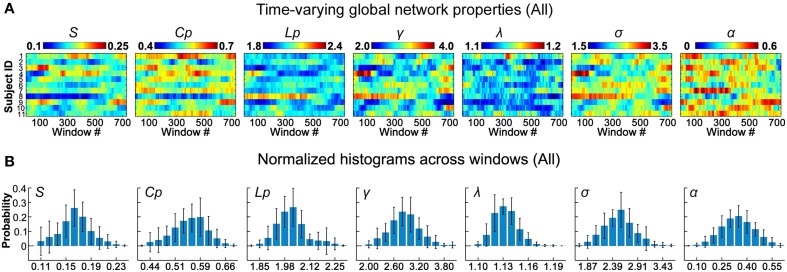
**Dynamic global topological properties of D-FC brain networks. (A)** Time-varying global network properties for all participants, including sparsity (*S*), clustering coefficient (*C*_*p*_), characteristic path length (*L*_*p*_), normalized clustering coefficient (γ), normalized characteristic path length (λ), small-worldness (σ), and assortativity (α). **(B)** Normalized histograms of global properties across windows for all participants. For each metric, a normalized histogram across windows was estimated for each participant; these histograms were then averaged across participants to generate the mean histogram. Error bars represent the standard deviation of the normalized histograms across participants. D-FC, dynamic functional connectivity.

To assess the time-varying topological properties of the functional networks, for each participant, we first generated a set of binary D-FC matrices over time using a thresholding approach (Bonferroni correction, *p*_*corr*_ < 0.01), and then performed graph-based network analyses. We showed that the global topological properties (*S, C*_*p*_, *L*_*p*_, γ, λ, σ, and α) of the D-FC brain networks fluctuated over time (Figure 2A and Table S3) and obtained corresponding normalized histograms across windows (Figure 2B). First, for all participants, network connectivity density or sparsity (*S*) fluctuated within a similar range (0.10–0.24), with the distribution centered at approximately 0.17. Out of the 8019 (i.e., 11 participants × 729 windows) D-FC networks obtained, 97.8% were fully connected, and the remaining networks had at least 88 nodes connected (Table S4). Second, all of the D-FC networks exhibited a clustering coefficient (*C*_*p*_) above 0.4 and a characteristic path length (*L*_*p*_) below 2.4. Compared to matched random networks, these D-FC networks were more locally clustered (i.e., γ = *C*${}_{p}^{brain}$*/C*${}_{p}^{rand}$ >> 1) and had an almost identical path length (i.e., λ = *L*${}_{p}^{brain}$*/L*${}_{p}^{rand}$ ~ 1) for all windows, indicating dynamic, but persistent small-world architecture over time (all σs > 1.6). Third, the temporal fluctuations of assortativity (α) had a distribution centered at approximately 0.35 (all α_*z*−*score*_ > 1.64), suggesting that nodes that possessed a similar number of edges tended to connect with each other.

### Dynamic hubs in functional brain networks

**Figure 3:**
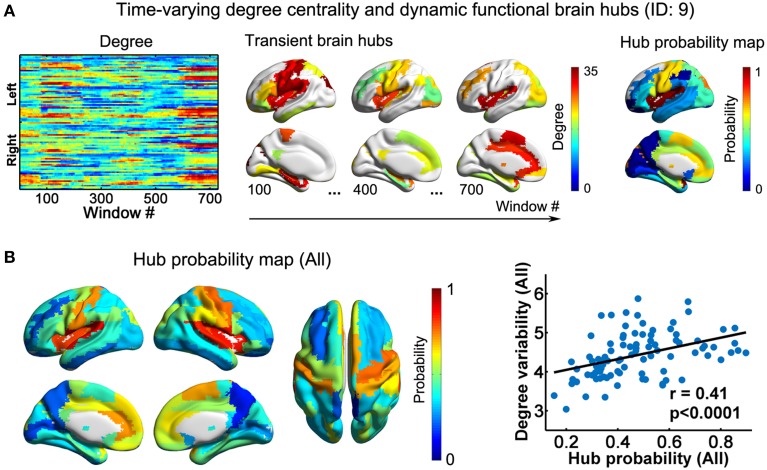
**Dynamic functional hubs in the D-FC brain networks. (A)** Time-varying degree centrality and dynamic functional brain hubs for one representative participant (ID: 9). Transient brain hubs at several windows (e.g., # = 100, 400, and 700) are displayed. Hub probability map was generated by counting the occurrence probability as hubs across sliding windows for each region. **(B)** Hub probability map for all participants (left) and scatter plot showing the across-node relationship between hub probability and temporal variability of functional degree centrality (right). The probability map for all participants was generated by averaging individual hub probability maps across participants. The surface-based visualizations were made using BrainNet Viewer (http://www.nitrc.org/projects/bnv/, Xia et al., 2013). D-FC, dynamic functional connectivity.

Using nodal degree centrality, we assessed the nodal roles in information communication of the D-FC networks. For each participant, the degree centrality of brain regions fluctuated over time, reflecting dynamic reconfiguration of spatial patterns (Figure 3A and Video S2 for one representative participant, ID: 9). Further, we identified transient functional hubs (above the mean) for each sliding window and estimated their occurrence probabilities across windows. For the participant with ID 9, we found that several regions appeared as persistent hubs across time with large probabilities (>0.5), which were mainly located at the insula, sensorimotor cortex, superior occipital gyrus, superior temporal gyrus, medial prefrontal cortex, and anterior and middle cingulate cortex (Figure 3A). At the group level, these regions were also identified as persistent functional hubs over time (Figure 3B, Table 1). Finally, the across-node correlation analysis revealed a significant positive association between the occurrence probability as hubs and the temporal variability of nodal degree centrality in the D-FC networks (*r* = 0.41, *p* < 0.0001; Figure 3B), indicating that functionally persistent hubs (with denser connectivity across time) tended to exhibit larger temporal variability.

**Table 1:** **Functionally persistent hubs in dynamic brain networks (AAL-90)**.

**ROI name**	**Hub probability**	**Degree variability (z-score)**
L Rolandic operculum	0.90	0.09
L Superior temporal gyrus	0.87	0.19
R Insula	0.85	1.18
R Rolandic operculum	0.85	0.51
R Superior temporal gyrus	0.84	0.10
L Insula	0.81	1.12
R Heschl gyrus	0.80	−0.22
R Precental gyrus	0.77	0.34
L Heschl gyrus	0.75	−0.03
L Postcentral gyrus	0.74	0.39
L Anterior cingulate and paracingulate gyri	0.73	0.29
R Supplementary motor area	0.71	0.62
R Postcentral gyrus	0.70	0.10
R Lenticular nucleus, putamen	0.67	1.90
R Temporal pole: superior temporal gyrus	0.67	2.34
L Superior frontal gyrus, medial	0.65	−1.10
R Anterior cingulate and paracingulate gyri	0.64	0.53
R Supramarginal gyrus	0.63	0.90
L Lenticular nucleus, putamen	0.63	0.64
L Temporal pole: superior temporal gyrus	0.62	1.42
L Gyrus rectus	0.60	−0.07
L Median cingulate and paracingulate gyri	0.60	1.14
R Superior frontal gyrus, medial	0.59	−1.12
L Superior occipital gyrus	0.58	−0.72
R Superior frontal gyrus, medial orbital	0.57	−1.28
L Superior frontal gyrus, medial orbital	0.57	−1.33
R Superior occipital gyrus	0.55	0.19
L Precental gyrus	0.54	0.44
L Paracentral lobule	0.54	1.08
R Median cingulate and paracingulate gyri	0.53	0.87
R Inferior frontal gyrus, opercular part	0.53	−0.04
L Inferior frontal gyrus, opercular part	0.53	0.62
R Gyrus rectus	0.52	0.35
L Angular gyrus	0.51	−1.09

*Functionally persistent hub regions (i.e., probability >0.5) at the group level are listed here. L, left; R, right. Hub probability represents the occurrence probability as functional hubs across windows, and degree variability denotes temporal variability of functional degree centrality across windows, which was further converted to z-score value by subtracting its mean across the brain and then dividing by the associated standard deviation. Positive z-score values indicate large temporal variability above the mean value across the brain*.

### Relationship between dynamic functional networks and structural connectivity networks

#### Connectivity relationship between D-FC and SC

**Figure 4:**
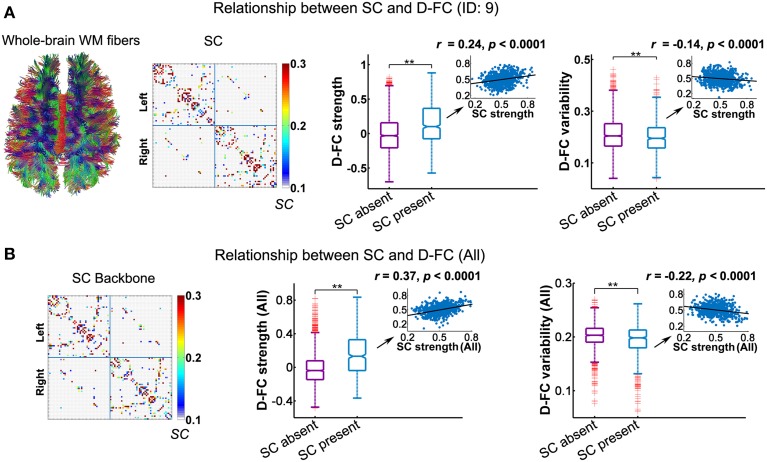
**Relationship between the D-FC and structural connectivity (SC) networks for one representative participant (ID: 9) (A) and for the whole group (B)**. **(A)** Schematic of whole-brain white matter (WM) fibers and SC matrix (left), and the differences in D-FC strength and variability between region pairs with direct SC (SC present) or not (SC absent) (right). The WM fibers were estimated from whole-brain tractography, and SC strength was represented by the normalized streamline number. **(B)** The SC backbone network across all participants (left) and differences in D-FC strength and variability between region pairs with or without direct SC (right). Group-level analyses were performed on the 10 participants that possessed both R-fMRI and DTI data, given that the DTI data of one participant (ID: 1) were not available. In **(A,B)**, significant differences in D-FC strength/variability between two groups were tested using permutation tests (^**^p < 0.0001, 10,000 permutations). Scatter plots (insets) show the correlation between D-FC strength/variability and SC strength, confined to the regional pairs with direct SC. Prior to the correlation analysis, all of these measures were resampled into a Gaussian distribution, with mean ± std = 0.5 ± 0.1. D-FC, dynamic functional connectivity.

Using DTI tractography, we reconstructed the white matter fibers in the whole brain for each participant, and then generated individual SC networks. Figure 4A displays the whole-brain white matter fibers and corresponding structural network for one representative participant (ID: 9). We found that regional pairs with direct SC exhibited significantly larger D-FC strength and lower D-FC temporal variability than those without direct SC (Figure 4A, both *ps* < 0.0001, 10,000 permutations). Moreover, for those region pairs with direct SC, D-FC strength was positively correlated with SC strength (*r* = 0.24, *p* < 0.0001), whereas D-FC variability was negatively correlated with SC strength (*r* = −0.14, *p* < 0.0001). Similar results were also observed for other participants (Table 2) and for the group-level analysis based on the SC backbone network (Figure 4B).

**Table 2:** **Relationship between dynamic functional connectivity and structural connectivity**.

**Participant ID**	**R_(D−FCstrength, SC)_**			**R_(D−FCvariability, SC)_**		
	**AAL-90**		**H-1024**	**AAL-90**		**H-1024**
	**Session 1**	**Session 2**	**Session 1**	**Session 1**	**Session 2**	**Session 1**
1	n.a.	0.27	n.a.	n.a.	−0.07	n.a.
2	0.33	0.30	0.20	−0.05^b^	−0.18	−0.13
3	0.28	0.25	0.15	−0.12	−0.14	−0.09
4	0.28	0.23	0.22	−0.18	−0.15	−0.15
5	0.25	0.29	0.21	−0.12	−0.07	−0.12
6^a^	0.27	0.26	0.21	−0.14	−0.17	−0.13
7	0.30	0.26	0.19	−0.08	−0.10	−0.12
8	0.23	0.33	0.18	−0.07	−0.16	−0.05
9	0.24	0.28	0.21	−0.14	−0.10	−0.14
10	0.28	0.27	0.21	−0.10	−0.10	−0.12
11	0.29	0.37	0.22	−0.17	−0.20	−0.13
Mean ± std	0.28 ± 0.03	0.28 ± 0.04	0.20 ± 0.02	−0.12±0.04	−0.14±0.05	−0.12±0.03

*For both low-resolution (AAL-90) and high-resolution (H-1024) brain networks, significantly different D-FC strength and variability were observed between region pairs with direct SC and those without direct SC for most participants (ps < 0.05, 10,000 permutations). R_(D−FC strength, SC)_ and R_(D−FC variability, SC)_ represent Pearson's correlations of SC strength vs. D-FC strength and D-FC variability, respectively, confined to region pairs with direct SC. Significant correlation (p < 0.05) was observed for almost all participants*.

*For Session 1, the structural and functional relationship was inferred for only 10 participants (ID: 2–11) that possessed both R-fMRI and DTI data. n.a denotes DTI data were not available*.

a *Significant difference in D-FC variability was not observed between region pairs with direct SC and those without direct SC (p > 0.05, 10,000 permutations) (ID: 6 in Session 1, in the AAL-90 brain networks)*.

b *D-FC variability did not exhibit significant correlation with SC strength (p > 0.05)*.

#### Topological relationship between D-FC and SC networks

**Figure 5:**
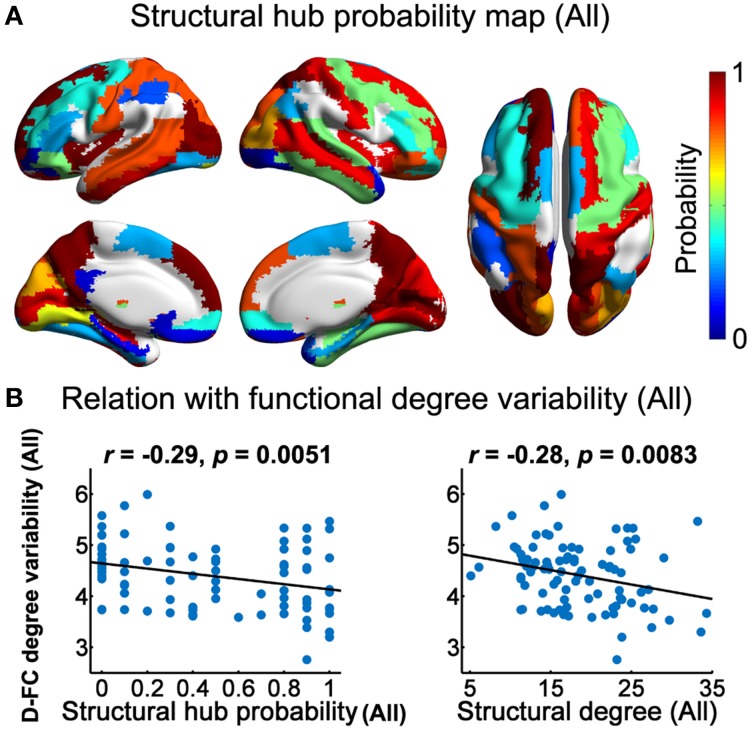
**Structural brain hubs and their associations with D-FC networks. (A)** Structural hub probability map for all participants, which was generated by counting the occurrence probability as structural hubs across participants for each region. **(B)** Scatter plots showing the across-node relationship between temporal variability of functional degree centrality in the D-FC networks and structural hub probability (left) and structural degree centrality (right). Given the discrete values of structural hub probability, Spearman's correlation analysis was used. D-FC, dynamic functional connectivity.

We calculated both global and nodal topological properties for individual binary SC networks and examined their associations with the temporal features of D-FC networks (e.g., temporal mean and variability of the topological properties across the sliding windows). First, we found that all of the individual SC networks exhibited small-world architecture (mean ± std: σ = 1.98 ± 0.13), characterized by dense local clustering (γ > 1) and short path length (λ ~ 1) (Table 3). Meanwhile, assortative organization was also observed for each participant, accompanied by α_*z*−*score*_ > 1.64 (one-tailed *p* < 0.05). Notably, for each global metric (*C*_*p*_, *L*_*p*_, γ, λ, σ, and α), its temporal mean and variability in D-FC networks did not exhibit significant across-subject correlation (*p*s > 0.05) with the SC networks. Second, for the SC networks of each participant, we identified the pivotal hub regions with larger degree centrality (above the mean). Figure 5A displays the probability map of structural hubs across individuals: structurally persistent hubs (probability >0.5) were mainly located in the insula, medial prefrontal cortex, sensorimotor cortex, precuneus, middle temporal gyrus, and visual cortex (Table 4). Notably, some of these regions also appeared as functionally persistent hubs (Figure 3B), including the insula, medial prefrontal cortex, sensorimotor cortex, and superior occipital gyrus. Further across-node correlation analysis revealed that both structural hub probability (*r* = −0.29, *p* = 0.0051) and structural degree centrality (*r* = −0.28, *p* = 0.0083) were negatively correlated with the temporal variability of functional degree centrality in the D-FC networks (Figure 5B).

**Table 3:** **Summary of global properties of individual structural brain networks (AAL-90)**.

**Participant ID**	***S***	***C_*p*_***	***L_*p*_***	**γ**	**λ**	**σ**	**α**
1	n.a.	n.a.	n.a.	n.a.	n.a.	n.a.	n.a.
2	0.19	0.53	1.78	2.18	1.05	2.08	0.02
3	0.19	0.54	1.86	2.41	1.09	2.21	0.06
4	0.21	0.55	1.77	2.06	1.06	1.95	0.02
5	0.22	0.52	1.72	1.90	1.04	1.83	0.04
6	0.22	0.58	1.76	2.12	1.06	1.99	0.02
7	0.22	0.54	1.75	2.05	1.05	1.95	0.06
8	0.20	0.55	1.81	2.26	1.07	2.11	0.01
9	0.20	0.55	1.78	2.15	1.06	2.03	0.04
10	0.22	0.56	1.74	2.03	1.05	1.92	0.01
11	0.21	0.54	1.73	1.82	1.04	1.75	0.03
Mean ± std	0.21 ± 0.01	0.55 ± 0.02	1.77 ± 0.04	2.10 ± 0.17	1.06 ± 0.01	1.98 ± 0.13	0.03 ± 0.02

*For each participant, we calculated global properties of the binary SC network. S indicates the network connectivity density or sparsity. C_p_, L_p_, γ, λ, σ, and α denote the clustering coefficient, characteristic path length, normalized clustering coefficient, normalized characteristic path length, small-worldness and assortativity, respectively. For all participants considered, the SC networks exhibited a small-world (σ > 1) and assortative (α > 0) architecture, with the assortativity coefficient significantly (α_z−score_ > 1.64) higher than those in random networks. Notably, only 10 participants (ID: 2–11) that possessed DTI data were included in structural network analysis. n.a. denotes DTI data were not available*.

**Table 4:** **Structurally persistent brain hubs across participants (AAL-90)**.

**ROI name**	**Hub probability**	**Structural degree centrality**
L Superior frontal gyrus, dorsolateral	1.00	27.10
L Superior frontal gyrus, medial	1.00	26.60
L Insula	1.00	23.10
R Calcarine fissure and surrounding cortex	1.00	23.80
L Middle occipital gyrus	1.00	27.70
R Superior parietal gyrus	1.00	26.20
L Precuneus	1.00	34.30
R Precuneus	1.00	33.60
L Lenticular nucleus, putamen	1.00	29.00
R Lenticular nucleus, putamen	1.00	33.20
R Superior frontal gyrus, dorsolateral	0.90	29.70
L Superior frontal gyrus, orbital part	0.90	23.80
R Insula	0.90	25.20
L Hippocampus	0.90	25.50
L Calcarine fissure and surrounding cortex	0.90	23.20
R Cuneus	0.90	25.00
R Lingual gyrus	0.90	27.50
R Postcentral gyrus	0.90	22.90
R Caudate nucleus	0.90	22.40
R Middle temporal gyrus	0.90	22.20
L Inferior temporal gyrus	0.90	22.70
R Superior frontal gyrus, orbital part	0.80	23.60
R Inferior frontal gyrus, orbital part	0.80	24.50
R Superior frontal gyrus, medial	0.80	22.80
L Superior occipital gyrus	0.80	23.10
R Superior occipital gyrus	0.80	23.70
L Postcentral gyrus	0.80	21.40
L Superior parietal gyrus	0.80	23.50
L Caudate nucleus	0.80	24.50
L Thalamus	0.80	24.90
L Middle temporal gyrus	0.80	22.30
L Cuneus	0.70	21.80
R Middle occipital gyrus	0.70	20.80
L Lingual gyrus	0.60	20.10

*Persistent structural hub regions across participants (i.e., probability >0.5) are listed here. L, left; R, right. Hub probability represents the occurrence probability as structural hubs across participants, and structural degree centrality denotes the average nodal structural degree values across participants*.

### Dynamic, high-resolution functional brain networks

**Figure 6:**
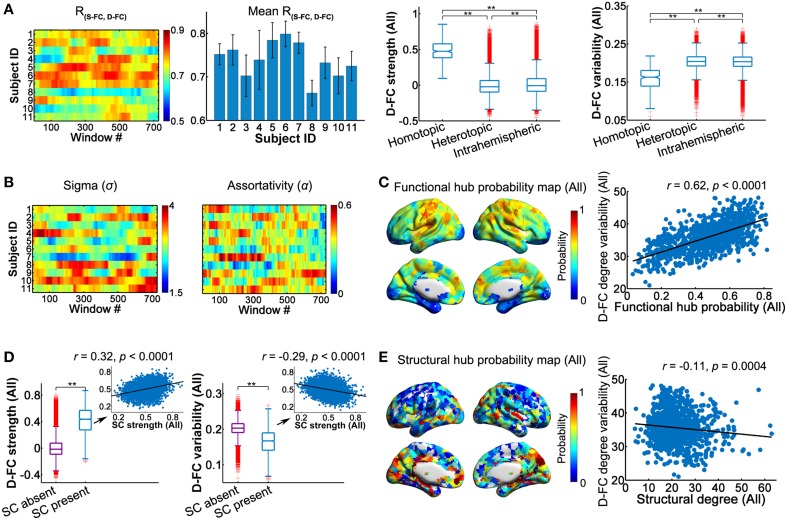
**Dynamic characteristics of high-resolution (H-1024) D-FC brain networks and their structural associations. (A)** Spatial similarity between the S-FC and the D-FC matrices over time (left) and the spatial-dependence of D-FC strength and variability for all participants (right). ^**^Bonferroni corrected *p* < 0.001, 10,000 permutations. **(B)** Time-varying small-worldness and assortativity of D-FC networks. **(C)** Functional hub probability map for all participants (left) and scatter plot showing across-node relationship between functional hub probability and the temporal variability of functional degree centrality in the D-FC networks (right). **(D)** Differences in D-FC strength (left) and temporal variability (right) between region pairs with or without direct SC. ^**^p < 0.0001. Scatter plots (insets) represent the across-connection relationship of D-FC strength (left) and D-FC variability (right) vs. SC strength, confined to the regional pairs with direct SC. Relevant statistical analyses were performed for all participants based on the SC backbone network. **(E)** Structural hub probability map for all participants (left) and across-node relationship with the temporal variability of functional degree centrality (right). The hub map was generated by counting the occurrence probability as structural hubs across participants for each region. SC, structural connectivity; S-FC, static functional connectivity; D-FC, dynamic functional connectivity.

Using the high-resolution regional parcellation (H-1024), we examined the temporal characteristics of the D-FC networks and relevant structural basis on a finer spatial scale (Figure 6 and Table S5). The main findings in the high-resolution networks were largely compatible with those observed in low-resolution (AAL-90) networks, which are outlined below: (i) *Spatial patterns of D-FC*. We found that the spatial patterns of the inter-regional D-FC in the high-resolution brain networks remained relatively stable over time (see Video S3 for one represent participant, ID: 9), with homotopic D-FC exhibiting the highest strength and lowest temporal variability (Figure 6A). (ii) *Topological properties of D-FC networks*. For all participants, network connectivity sparsity (*S*) fluctuated within a similar range (0.05–0.15), with the distribution centered at approximately 0.10. Out of the 8019 (i.e., 11 participants × 729 windows) D-FC networks obtained, 98.5% were fully connected, and the remaining networks had 1023 nodes connected (Table S4). We found that individual high-resolution D-FC networks exhibited time-varying, but persistent small-world and assortative architecture (Figure 6B), and possessed functional brain hubs that persisted over time (probability >0.5) (Figure 6C, and Video S4 for one representative participant, ID: 9). Notably, the spatial distribution of persistent hubs in the high-resolution D-FC networks was largely consistent with the distribution in the low-resolution brain networks, except that additional functional hubs were observed in the high-resolution D-FC networks in the posterior cingulate cortex and precuneus. (iii) *Relationship between the D-FC and SC networks*. First, at the connection level, region pairs with direct SC showed significantly greater D-FC strength and smaller D-FC variability than those without SC (both *p*s < 0.0001, 10,000 permutations; Figure 6D). Moreover, for structurally connected regions, SC strength exhibited significant correlations with both D-FC strength (*r* = 0.32) and variability (*r* = −0.29; Figure 6D, Table 2). Second, at the global topological level (small-world and assortativity properties), significant across-subject correlation was not observed (*ps* > 0.05) between individual SC networks and associated temporal features of D-FC networks. Finally, at the regional level, several structural brain hubs across individuals also appeared as functionally persistent hubs over time, such as the insula, medial prefrontal gyrus, somatosensory cortex, superior occipital gyrus, and precuneus (Figure 6E). Moreover, structural hubs with larger SC degree centrality tended to have lower D-FC degree variability across time (*r* = −0.11; Figure 6E).

### Validation results

We validated our results by using different scanning sessions and different analysis strategies (involving the window lengths, correlation thresholding strategies and network types). (i) *The effects of different scanning session*. We found that most of the findings reported above were highly reproducible using the multiband R-fMRI and DTI data obtained from the same participants during the second scanning session, which was separately from the first by approximately 1 week (Table 2, Figure S1). (ii) *The effects of window length*. We re-generated the individual D-FC networks using different window lengths (window length = 50 or 150 s) and found that the temporal features of the functional organization, including the connections, global properties and nodal degree, were highly similar to our main results (Figures S2, S3). Moreover, the connectivity strength and temporal variability of the D-FC were significantly correlated with SC strength at different window lengths, but the relationship was more significant when a smaller window length was used (Figure S2D vs. Figure S3D). (iii) *The effects of correlation thresholding strategy*. We found that the main results were preserved when different correlation thresholding strategies were used for D-FC network construction, including the varying Bonferroni correction thresholds (*p*_*corr*_ < 0.05 or 0.001; Figures S4, S5), and different network sparsity values (*S* = 10, 15, and 20%; Figure S6). (iv) *The effects of network type*. We regenerated weighted D-FC networks, and found that retaining the edge weights during the brain network analyses did not change our main findings (Figure S7).

## Discussion

Using the sub-second multiband R-fMRI and DTI data, we comprehensively explored the dynamic topological organization of human whole-brain functional networks at a finer temporal resolution (TR = 645 ms) and provided the first demonstration of their underlying structural substrates. The main results were summarized as follows: (i) The overall D-FC patterns maintained high spatial similarity with the static connectivity pattern, with larger connectivity strength and lower temporal variability in homotopic D-FC than heterotopic and intrahemispheric D-FC. The D-FC networks exhibited time-varying but persistent small-worldness, assortativity and functional hubs, with several regions (e.g., insula, sensorimotor cortex, and medial prefrontal cortex) emerging as functionally persistent hubs but having larger temporal variability in degree centrality than other regions. (ii) The temporal characteristics (i.e., strength and variability) of the D-FC were significantly associated with the existence and the strength of structural connections. The temporal variability of nodal degree centrality in the D-FC networks was anatomically constrained by the structural networks. Collectively, these results provide novel insights into the spontaneous or intrinsic network dynamics of the human brain and their underlying structural substrates at a finer time scale (seconds).

### Dynamic functional connectivity patterns in the resting human brain

Traditional static FC analysis using the whole-scan time courses has greatly advanced our understanding of the intrinsic functional organization of the brain (Biswal et al., 1995; Fox and Raichle, 2007; Kelly et al., 2012). However, this approach cannot capture the time-varying characteristics of functional organization. Using a sliding window approach, in the present study, we observed that functional interactions among regions during rest underwent spontaneous dynamic reorganization (Figure 1, Videos S1, S3). On the one hand, these diverse D-FC patterns may be due to the shift of arousal states during the unconstrained resting scanning (Allen et al., 2014). On the other hand, and more possibly, the spontaneous fluctuations of D-FC patterns may be the manifestation of intrinsic brain dynamics (Hutchison et al., 2013b). Recent empirical (Tagliazucchi et al., 2012a; Liu and Duyn, 2013) and theoretical (Deco et al., 2011, 2013; Haimovici et al., 2013) studies suggest that the resting brain may reside near a critical state, supporting a rich repertoire of functional coordination patterns. Thus, the switch of large-scale D-FC patterns may reflect the exploration of available functional configurations, which would benefit the efficiency and the speed for the configuration selection in response to potential cognitive demands (Deco et al., 2013). Interestingly, previous electrophysiological studies (Kramer et al., 2011; Chu et al., 2012) with long-term recordings (~ days) in the human brain found that a network template emerged from short time intervals (~ 100 s) underlying the dynamic functional organization. Here, we provide evidence that the large-scale D-FC patterns derived from R-fMRI data persistently exhibited high spatial similarity with the static pattern (Figures 1C, 6A), indicating that the latter might serve as a template on which diverse, transient functional configurations emerge and recede with minor modulations. We assume that the template for functional coordination may be manifestation of the influence of anatomical architecture, the detection and the physiological meaning of which require further elucidation.

### Dynamic small-world and assortative functional brain networks

Small-world brain networks serve as an attractive model for brain organization, because they supports efficient information segregation and integration through dense local clustering and yet short path length between any pair of nodes (Watts and Strogatz, 1998). A number of studies have consistently found small-world architecture in static functional brain networks across diverse imaging modalities (e.g., fMRI, EEG, and MEG) (for reviews, see Bassett and Bullmore, 2006; Bullmore and Sporns, 2009, 2012; He and Evans, 2010), suggesting that it is a general principle of brain organization. Using a sliding window approach, we observed that although the measure of small-worldness fluctuated substantially over time, small-world topology was preserved across all of the windows in company with low sparsity (i.e., wiring costs; Figures 2, 6B), indicating that the human brain maintains a dynamic balance between efficient local and global communications to meet changeable environments. More specifically, although the functional connections attached to each node were variable across time (Figure 3A), the functional connection patterns across the whole brain maintained significant assortativity (Figures 2, 6B). As a consequence, high-degree hub nodes preferentially connected with each other, constituting a relatively resilient “connectivity core.” Assortative topology has been readily observed in static functional networks of the human brain (Eguíluz et al., 2005; Park et al., 2008; Braun et al., 2012). Maintaining this feature during dynamic organization could be beneficial for continual information processing among hubs, and could thus promote a fast response to changing cognitive demands and increase the resilience to potential pathological attacks.

### Persistent but flexible functional brain hubs

We found that brain regions exhibited substantial fluctuations across time in their nodal degree centrality values (Figure 3A, Videos S2, S4), indicating their variable functional roles at a short time scale. Of note, several regions emerged as functionally persistent hubs (i.e., >50% of the windows) over time (Figures 3B, 6C), which were mainly located in the sensorimotor cortex, default-mode network (e.g., medial prefrontal cortex, angular gyrus, posterior cingulate gyrus and precuneus) and the cingulo-opercular control system (e.g., anterior cingulate cortex and insula). The majority of these regions have been identified as functional hubs in previous static network studies (Achard et al., 2006; Buckner et al., 2009; Liang et al., 2013; Liao et al., 2013; van den Heuvel and Sporns, 2013) and are supposed to be crucial for efficient functional coordination across spatially distributed regions. Notably, our findings highlight their enduring contributions to global information integration and provide insights into their functional roles in dynamic network integrity. Interestingly, previous studies of the human brain (Allen et al., 2014; Zalesky et al., 2014) suggest that functional connectivity involving these regions is highly variable over time. Here, using quantitative degree centrality analysis, we demonstrated that the functionally persistent hubs tended to exhibit larger temporal variability in functional degree centrality than other regions, regardless of the spatial resolution considered (Figures 3B, 6C). Interestingly, highly variable functional hubs have also been observed in macaque cortical networks using both simulation (Honey et al., 2007) and empirical (Shen et al., 2015) data, in which functional hubs were estimated from longer time periods (~ minutes). In a task-based fMRI study, Cole et al. (2013) pointed out that the flexible fronto-parietal hub regions may update their functional connectivity patterns rapidly according to different task states. Using multiband R-fMRI, we further demonstrated the dynamic characteristics of functional brain hubs during rest, including their persistence and flexibility across time. These persistent hubs dynamically adjust their global functional connections involving multiple systems, to enable adaptive information exchange and integration.

### Structural substrates of dynamic functional organization

Simulations of macaque brain networks have shown that inter-regional functional connectivity at multiple time scales (e.g., seconds to minutes) is shaped by the anatomical architecture (Honey et al., 2007). Very recently, using R-fMRI in macaques Shen et al. (2015) provided an empirical demonstration that more stable functional connections are observed between region pairs with direct anatomical connections obtained from axonal tract tracing. However, for the human brain, the majority of studies have focused mainly on the structural substrates for static functional connectivity or networks, and the structural constraints on spontaneous evolution of functional networks are still unclear. Based on multiband R-fMRI and DTI data of the same participants, we provided crucial evidence for the structural substrates underlying the large-scale dynamic functional organization of the human brain on the order of seconds, at both connection and network levels.

At the connection level, we found that both the connectivity strength and the temporal variability of D-FC were dependent on the spatial interrelationships (Figures 1D, 6A), which agrees well with previous studies on S-FC (Stark et al., 2008) and D-FC that fluctuates on a scale of minutes (Gonzalez-Castillo et al., 2014). One possible reason is that as homologous regions possess similar functional roles (Toro et al., 2008; Crossley et al., 2013), homotopic FC between them may manifest as continual functional interaction, with less temporal variation. In contrast, heterotopic and intrahemispheric FC usually connects regions involved in specialized functions, yielding occasional inter-regional functional coupling with larger temporal variability. Moreover, the direct SC, corpus callosum, linking homologous regions (Gong et al., 2009; van den Heuvel et al., 2009) may promote the temporal stability of homotopic FC. Although prior studies have shown that SC may shape the functional interactions or S-FC (Hagmann et al., 2008; Honey et al., 2009; van den Heuvel et al., 2009; Goñi et al., 2014), the structural influences on the temporal characteristics of functional couplings are usually ignored. Here, we highlighted that D-FC fluctuating on a scale of seconds was shaped by the underlying SC: the larger the structural connectivity strength, the greater the functional coupling with less temporal variability (Figures 4, 6D and Table 2), which extends our understanding of the structure-function relationships in the human brain at shorter time scales.

At the network level, we found that the dynamic functional networks and structural networks shared common global properties (e.g., small-worldness and assortativity; Figure 2 and Table 3), which was compatible with prior static FC studies (Bullmore and Sporns, 2009, 2012). Moreover, we observed that regions that possessed larger structural degree centrality or structural hub probability tended to exhibit less temporal variability of functional degree centrality, which could be due to the structural constraints on temporal variability of D-FC (Figure 4), as mentioned above. Of note, this negative relationship between structural degree centrality and the temporal variability of functional degree centrality is different from that for functional hubs, which may result from the different spatial patterns of the probability maps observed for functional hubs (Figure 3B) and structural hubs (Figure 5A). Although several hub regions in common (e.g., insula, medial prefrontal cortex) were found, we also observed that several structural hub regions (e.g., middle temporal gyrus and visual cortex) exhibited small probability as functional hubs, and several functionally persistent hubs (e.g., anterior cingulate cortex) possessed small structural degree centrality. These discrepancies in nodal centrality may be partially attributed to the fact that strong D-FC can also exist between region pairs without direct SC, as illustrated by the red outliers in Figure 4B (middle panel). As suggested by several static FC studies (Honey et al., 2009; Adachi et al., 2012), this unexpected D-FC could be mediated by indirect SC, making the inference of functionally persistent hubs from structural hubs via one-to-one mapping unfeasible. Additionally, the estimation of global network topology involves the large-scale connectivity patterns across the whole brain; thus the quantitative correspondence between structural and dynamic functional networks becomes difficult.

Recently, based on empirical SC data, several computational model studies point out that not only the anatomical structure but also the dynamics of local regions can affect the inter-regional functional coupling (Honey et al., 2009; Deco and Jirsa, 2012; Deco et al., 2013; Hansen et al., 2015). Through an enhanced non-linear mean-field model, Hansen et al. (2015) simulated the brain activities at a subcritical regime, and reproduced the spontaneous switches of FC states. These studies suggest that the behavior of D-FC cannot be understood in terms of SC alone, and incorporating computational models in future studies can obtain mechanistic insights into the complex structure-function relationship in the brain.

## Further considerations

Several issues should be considered further. First, the limited number of participants used for this study (11 participants) may restrict the statistical power of the results. Although the main results were replicated here by using repeated dataset (two sessions) of the same participants acquired around 1 week apart, it would be worthy to replicate the present findings based on a larger dataset in the future. Second, to capture the dynamics of functional networks, a commonly used sliding window approach was used here. However, to date, there is no universally accepted criterion for window selection (Hutchison et al., 2013a). To ensure the robustness of our results, three window lengths, 50, 100, and 150 s, were considered in the current study. Because, increasing attention has been paid to the time-varying functional organization of the brain (Hutchison et al., 2013a; Calhoun et al., 2014), a methodological framework for the choice of more appropriate windows (e.g., window type and window length) should be established with future efforts. Third, we found some differences across different parcellation schemes considered, such as additional functional hubs in the posterior cingulate cortex and precuneus in the high-resolution (H-1024) networks. These discrepancies could be due to that some specific ROIs (e.g., precuneus) in the AAL-90 parcellation are functionally inhomogeneous. Thus, the potential influence of different parcellation schemes on evaluating functional network dynamics deserves further investigation. Fourth, previous studies have suggested that head motion may introduce artificial functional connections (Power et al., 2012; Van Dijk et al., 2012; Satterthwaite et al., 2013). Several motion correction strategies have been recently proposed, including the widely used scrubbing approach (Power et al., 2012). Notably, data scrubbing involves the removal or interpolation of contaminated time points, which may damage the temporal continuity of time courses and thereby affect the estimation of D-FC patterns. Therefore, in the present study, we used a 24-parameter autoregression model (Friston et al., 1996) during data preprocessing to partially reduce the motion-induced artifacts while maintaining the temporal continuity of data. Future studies of developing new motion correction strategies are important to evaluate the potential influence of head motion on dynamic functional networks. Fifth, we employed a sub-second (TR = 645 ms) multiband R-fMRI technique for brain network dynamics, which captures fine temporal information of BOLD activities and partially removes the influence of respiratory signals (~0.3 Hz). Further works using advanced imaging protocols with higher sampling rates and simultaneous physiological signal recordings would be important to better infer the brain dynamics and further diminish the effects of non-neural signals (e.g., cardiac rhythms). Finally, we showed the dynamic features of functional brain networks and their structural correlates. These patterns could be modulated by different cognitive tasks and during normal development, aging and neuropsychiatric disorders, all of which are of interested for future studies.

### Conflict of interest statement

The authors declare that the research was conducted in the absence of any commercial or financial relationships that could be construed as a potential conflict of interest.
