# Supplementary material for: Spontaneous functional network dynamics and associated structural substrates in the human brain
Source: Front Hum Neurosci. 2015 Sep 4;9:478. doi: 10.3389/fnhum.2015.00478 (PMC4559598; doi:10.3389/fnhum.2015.00478)
Supplement: Supplementary file 5 [file Presentation1.PDF]

Supplementary Materials

Table S1. Summary of phenotype information of the participants

ID	Identifier Number	Sex	Age(y)	Current Diagnosis (n.a., not available)	Lifetime Diagnosis (n.a., not available)	Used
1	1427581	F	27	Healthy	Healthy	Yes[a]
2	2475376	M	21	Healthy	Healthy	Yes
3	2799329	M	30	Healthy	Healthy	Yes
4	2842950	M	27	Healthy	Healthy	Yes
5	3201815	M	48	Healthy	Healthy	Yes
6	3315657	M	19	Healthy	Healthy	Yes
7	3808535	M	25	Healthy	Healthy	Yes
8	4176156	M	46	Healthy	Healthy	Yes
9	7055197	F	22	Healthy	Healthy	Yes
10	8735778	F	31	Healthy	Healthy	Yes
11	9630905	F	36	Healthy	Healthy	Yes
12	3795193	M	57	Healthy	Healthy	No[b]
13	21001	M	57	Healthy	Healthy	No[c]
14	1793622	M	60	Healthy	305- Alcohol Abuse; 305.2- Cannabis Abuse;	No
15	1961098	F	21	296.20- Major Depressive Disorder, Single Episode, Unspecified; 305.2- Cannabis Abuse;	307.5- Eating Disorder NOS	No
16	3313349	F	22	Healthy	296.26- Major Depressive Disorder, Single Episode, Full Remission	No
17	3893245	M	38	296.35- Major Depressive Disorder, Recurrent, In partial remission	305- Alcohol Abuse	No
18	4288245	M	22	Healthy	305- Alcohol Abuse; 304.3- Cannabis Dependence; 311- Depressive Disorder NOS	No
19	6471972	M	32	300.02-Generalized Anxiety;	303.9-Alcohol Dependence, unspecified	No
20	8574662	M	42	296.31- Major Depressive Disorder, Recurrent, Mild; 300.23- Social Phobia;	305- Alcohol Abuse; 305.2- Cannabis Abuse; 304.2- Cocaine Dependence; 304- Opiod Dependence; 314.01- ADHD Combined Type	No
21	21002	M	52	n.a.	n.a.	No
22	21006	M	32	n.a.	n.a.	No
23	21018	M	36	n.a..	n.a.	No
24	21024	M	22	n.a.	n.a.	No

The diagnostic information for each participant was collected using structured clinical interview for DSM Disorder (SCID) by trained professionals and the numbers are DSM-IV codes. ‘Yes’ or ‘No’ indicate participants included in the current study or not.

[a] DTI data in Session 1 was not available.

[b] Participant was excluded due to excessive head motion (> 2mm or > 2°) in Session 2.

[c] Participant was excluded due to obvious brain atrophy.

Table S2. Regions of interest from AAL template (AAL-90)

Index	Region	Index	Regions
1,46	Precental gyrus	24,69	Lingual gyrus
2,47	Superior frontal gyrus, dorsolateral	25,70	Superior occipital gyrus
3,48	Superior frontal gyrus, orbital part	26,71	Middle occipital gyrus
4,49	Middle frontal gyrus	27,72	Inferior occipital gyrus
5,50	Middle frontal gyrus, orbital part	28,73	Fusiform gyrus
6,51	Inferior frontal gyrus, opercular part	29,74	Postcentral gyrus
7,52	Inferior frontal gyrus, triangular part	30,75	Superior parietal gyrus
8,53	Inferior frontal gyrus, orbital part	31,76	Inferior parietal, but supramarginal and angular gyri
9,54	Rolandic operculum	32,77	Supramarginal gyrus
10,55	Supplementary motor area	33,78	Angular gyrus
11,56	Olfactory cortex	34,79	Precuneus
12,57	Superior frontal gyrus, medial	35,80	Paracentral lobule
13,58	Superior frontal gyrus, medial orbital	36,81	Caudate nucleus
14,59	Gyrus rectus	37,82	Lenticular nucleus, putamen
15,60	Insula	38,83	Lenticular nucleus, pallidum
16,61	Anterior cingulate and paracingulate gyri	39,84	Thalamus
17,62	Median cingulate and paracingulate gyri	40,85	Heschl gyrus
18,63	Posterior cingulate gyrus	41,86	Superior temporal gyrus
19,64	Hippocampus	42,87	Temporal pole: superior temporal gyrus
20,65	Parahippocampal gyrus	43,88	Middle temporal gyrus
21,66	Amygdala	44,89	Temporal pole: middle temporal gyrus
22,67	Calcarine fissure and surrounding cortex	45,90	Inferior temporal gyrus
23,68	Cuneus		

The regions of interest are defined based on the prior automated anatomical labeling atlas (Tzourio-Mazoyer et al., 2002). For better visualization of the intra- and inter-hemispheric connectivities, regions in left and right hemispheres are reindexed separately, with regions in left hemisphere ranging from 1-45 and those in right hemisphere from 46-90.

Table S3. Summary of global properties of human brain dynamic functional networks (AAL-90)

Participant ID	S	C_p	L_p	γ	λ	σ	α
1	0.18±0.02	0.60±0.03	1.99±0.04	2.83±0.28	1.15±0.01	2.47±0.26	0.32±0.10
2	0.15±0.01	0.52±0.04	2.04±0.05	2.89±0.23	1.12±0.01	2.57±0.21	0.35±0.09
3	0.18±0.02	0.57±0.03	1.96±0.06	2.63±0.38	1.13±0.01	2.32±0.32	0.36±0.09
4	0.18±0.03	0.58±0.04	2.00±0.10	2.92±0.50	1.14±0.01	2.57±0.43	0.33±0.08
5	0.16±0.02	0.55±0.03	2.03±0.06	2.84±0.27	1.14±0.01	2.51±0.24	0.33±0.07
6	0.16±0.01	0.54±0.03	2.03±0.04	2.99±0.18	1.13±0.01	2.65±0.17	0.28±0.08
7	0.17±0.01	0.59±0.02	2.00±0.04	3.01±0.21	1.14±0.01	2.64±0.18	0.39±0.11
8	0.13±0.02	0.48±0.03	2.14±0.10	3.08±0.44	1.13±0.01	2.73±0.38	0.28±0.09
9	0.18±0.02	0.57±0.04	1.96±0.07	2.55±0.31	1.13±0.01	2.27±0.28	0.38±0.09
10	0.16±0.02	0.53±0.03	2.02±0.07	2.75±0.31	1.13±0.01	2.44±0.27	0.36±0.09
11	0.16±0.01	0.56±0.04	2.04±0.05	2.88±0.25	1.14±0.01	2.53±0.21	0.39±0.06

Dynamic functional networks were constructed using a sliding window approach, with window length = 155 TR (i.e., 100s) and sliding step = 1TR. S indicates the sparsity of the functional network thresholded with Bonferroni correction ($p_{corr} < 0.01$). C_p, L_p, γ, λ, σ and α denote the clustering coefficient, characteristic path length, normalized clustering coefficient, normalized characteristic path length, small-worldness and assortativity, respectively. For all global network properties, their temporal mean and temporal variability (i.e., standard deviation) across 729 sliding windows are listed. For each participant, the dynamic functional networks exhibited time-varying but robust small-world (σ > 1) and assortative (α > 0) architecture.

Table S4. Summary of the time-averaged size of the largest component of dynamic functional networks

Participant ID	Session 1 (AAL-90)								Session 1 (H-1024)	Session 2 (AAL-90)
	Bonferroni corrected *p*			Window length		Sparsity			Bonferroni corrected *p*	Bonferroni corrected *p*
	<0.01	<0.05	<0.001	50 s	150 s	10%	15%	20%	<0.01	<0.01
1	90.00	90.00	89.95	89.63	90.00	86.64	89.84	90.00	1023.91	89.98
2	89.98	90.00	89.91	89.67	90.00	89.40	89.96	90.00	1024.00	90.00
3	89.95	89.99	89.90	89.74	90.00	88.67	89.86	89.98	1024.00	89.99
4	89.96	89.97	89.92	89.85	90.00	89.37	89.95	89.98	1023.99	89.99
5	89.93	89.99	89.84	89.58	89.98	88.97	89.84	89.99	1023.99	89.99
6	89.99	90.00	89.95	89.66	90.00	89.43	89.94	90.00	1023.98	89.92
7	90.00	90.00	89.99	89.66	90.00	88.91	89.99	90.00	1024.00	90.00
8	89.98	90.00	89.88	89.60	89.99	89.74	90.00	90.00	1023.97	89.95
9	89.98	90.00	89.97	89.58	90.00	87.87	89.94	90.00	1024.00	89.98
10	89.97	89.99	89.92	89.68	90.00	89.39	89.97	90.00	1024.00	89.97
11	90.00	90.00	89.94	89.78	90.00	88.91	89.99	90.00	1024.00	89.93

Dynamic functional networks were constructed using a sliding window approach, which shifted in time with a step of one TR (i.e., 645 ms). In different network construction strategies, the sliding window length was set as 155 TRs (i.e., 100 s), if not mentioned otherwise. For each participant, we counted the sizes of the largest component of dynamic functional networks within each sliding window, and then averaged them across all sliding windows. The time-averaged sizes of the largest component of dynamic functional networks are listed for different network construction approaches.

Table S5. Summary of global properties of whole-brain structural networks (H-1024)

Participant ID	S	C_p	L_p	γ	λ	σ	α
1	n.a.	n.a.	n.a.	n.a.	n.a.	n.a.	n.a.
2	0.02(1023)	0.41	3.02	14.51	1.22	11.90	0.11
3	0.02(1020)	0.39	3.12	13.74	1.25	11.01	0.16
4	0.02(1022)	0.40	2.99	11.92	1.23	9.67	0.23
5	0.02(1024)	0.39	3.06	13.72	1.23	11.16	0.21
6	0.02(1024)	0.41	2.95	12.46	1.24	10.06	0.18
7	0.02(1024)	0.40	3.00	13.44	1.23	10.88	0.16
8	0.02(1022)	0.39	2.99	12.89	1.23	10.52	0.15
9	0.02(1024)	0.41	3.02	13.78	1.24	11.13	0.20
10	0.02(1024)	0.40	2.92	11.74	1.22	9.61	0.15
11	0.02(1023)	0.40	2.98	12.90	1.23	10.52	0.20
mean±std	0.02±0.00	0.40±0.01	3.01±0.06	13.11±0.89	1.23±0.01	10.65±0.72	0.18±0.04

For each participant, we calculated global properties for the high-resolution binary structural brain network. S indicates the network density or sparsity, and the number in the bracket indicates the size of the maximal component in the network. C_p, L_p, γ, λ, σ and α denote the clustering coefficient, characteristic path length, normalized clustering coefficient, normalized characteristic path length, small-worldness and assortativity, respectively. For all participants considered, the structural networks exhibited a small-world (σ > 1) and assortative (α > 0) architecture, with the assortativity coefficient significantly ($\alpha_{z\text{-}score}$ > 1.64) higher than those in random networks. Notably, only 10 participants (ID: 2-11) that possessed DTI data were included in structural network analysis. n.a. denotes DTI data were not available.

Supplementary Figures

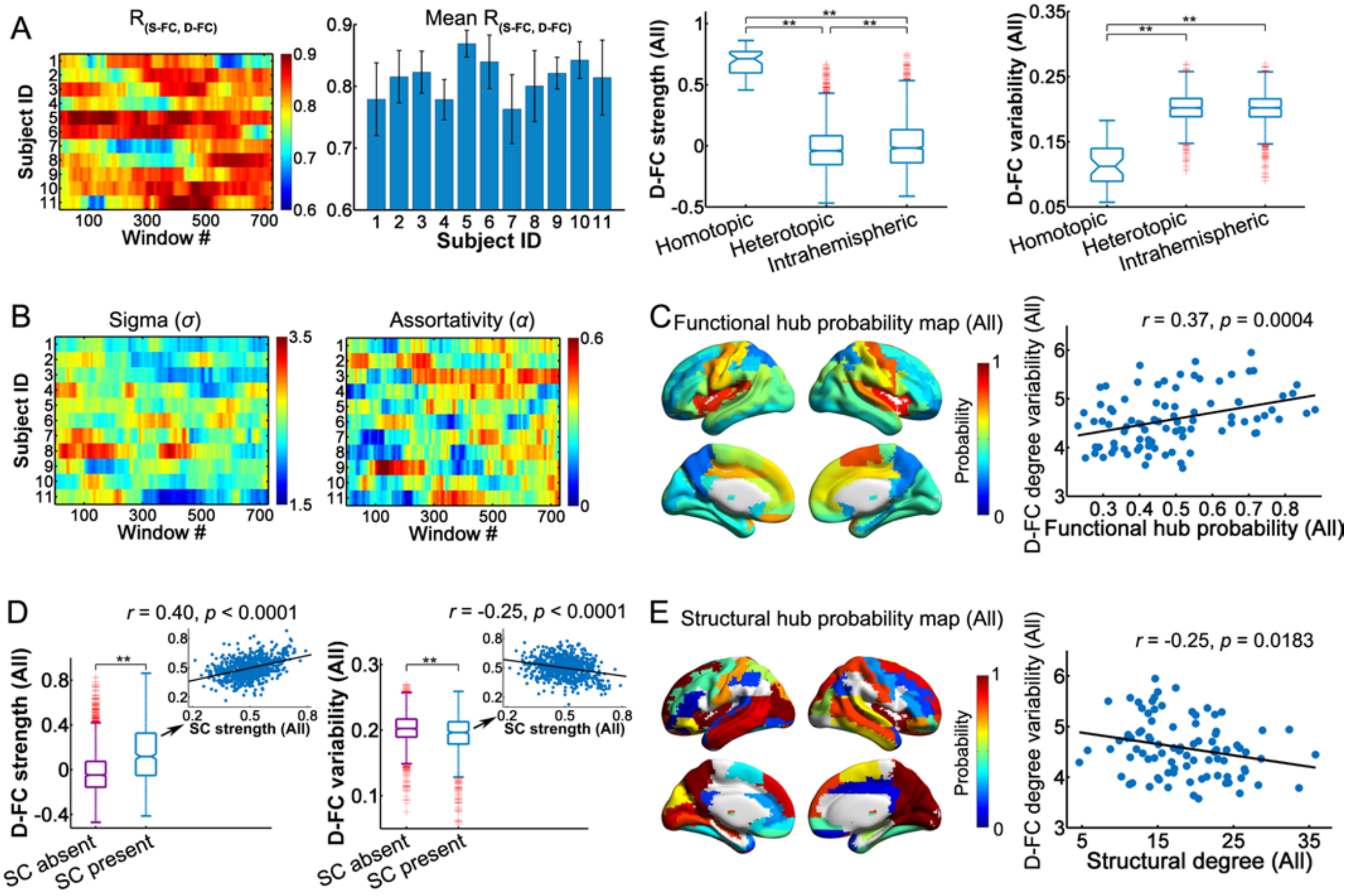

Figure S1. Dynamic characteristics of D-FC brain networks and their structural associations in Session 2. (A) Spatial similarity between the S-FC and the D-FC matrices over time (left) and the spatial-dependence of D-FC strength and variability for all participants (right). ** Bonferroni corrected $p < 0.001$, 10,000 permutations. (B) Time-varying small-worldness and assortativity of D-FC networks. (C) Functional hub probability map for all participants (left) and scatter plot showing across-node relationship between functional hub probability and the temporal variability of functional degree centrality in the D-FC networks (right). (D) Differences in D-FC strength (left) and temporal variability (right) between region pairs with or without direct SC. ** $p < 0.0001$, 10,000 permutations. Scatter plots (insets) represent the across-connection relationship of D-FC strength (left) and D-FC variability (right) versus SC strength, confined to the regional pairs with direct SC. Relevant statistical analyses were performed for all participants based on the SC backbone network. (E) Structural hub probability map for all participants (left) and across-node relationship with the temporal variability of functional degree centrality (right). The hub map was generated by counting the occurrence probability as structural hubs across participants for each region. Notably, brain network analyses here were performed in Session 2 based on AAL-90 atlas, and individual D-FC networks were obtained using a sliding window approach with a window length = 100 s followed by a Bonferroni correction ($p_{corr} < 0.01$). SC: structural connectivity; S-FC: static functional connectivity; D-FC, dynamic functional connectivity; AAL-90, automated anatomical labeling atlas. The surface-based visualizations were made using BrainNet Viewer (http://www.nitrc.org/projects/bnv/, Xia et al. 2013), same hereinafter.

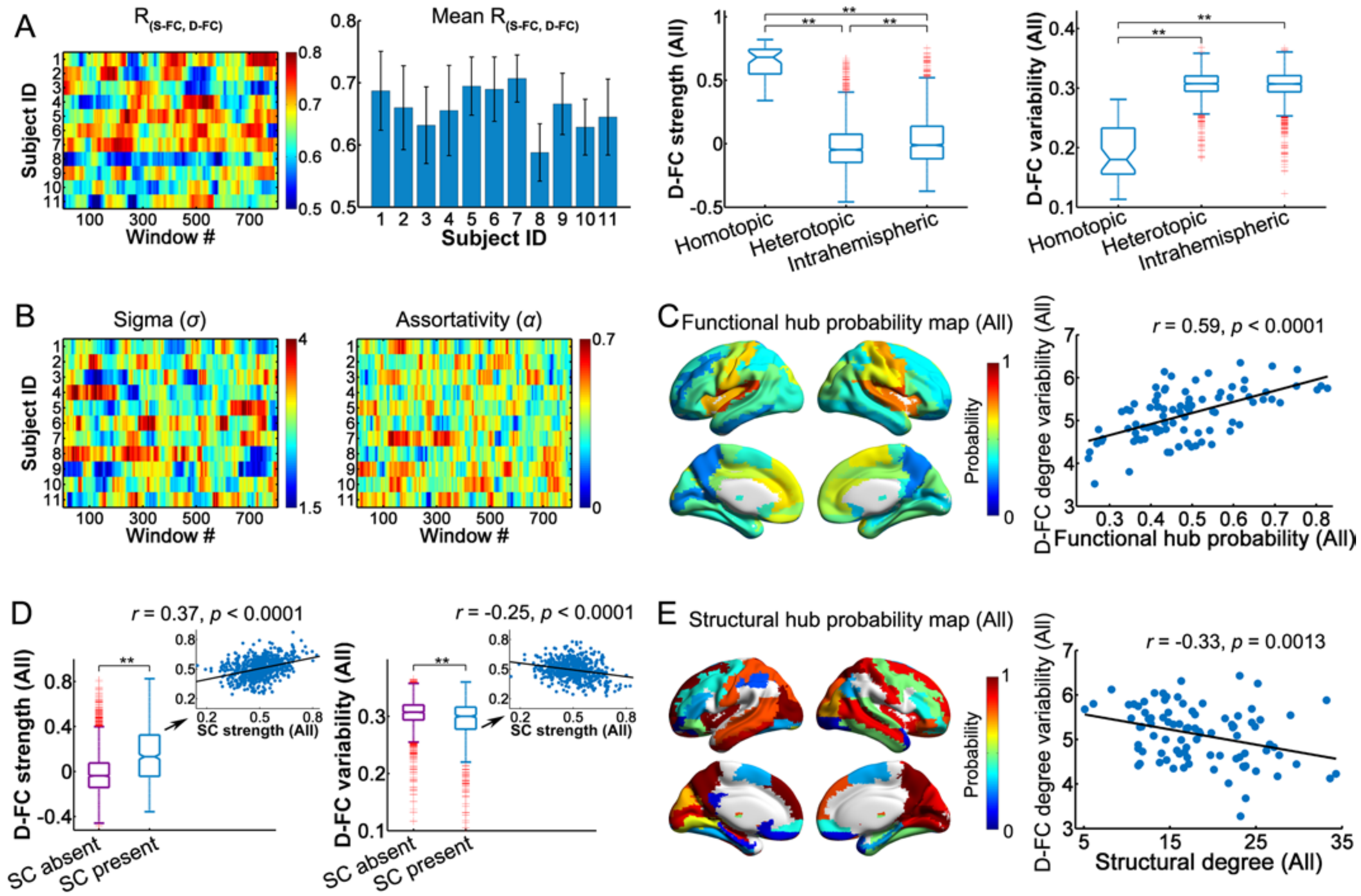

Figure S2. Dynamic characteristics of D-FC brain networks with a window length of 50 s and their structural associations. (A) Spatial similarity between the S-FC and the D-FC matrices over time (left) and the spatial-dependence of D-FC strength and variability for all participants (right). ** Bonferroni corrected p < 0.001, 10,000 permutations. (B) Time-varying small-worldness and assortativity of D-FC networks. (C) Functional hub probability map for all participants (left) and scatter plot showing across-node relationship between functional hub probability and the temporal variability of functional degree centrality in the D-FC networks (right). (D) Differences in D-FC strength (left) and temporal variability (right) between region pairs with or without direct SC. ** p < 0.0001, 10,000 permutations. Scatter plots (insets) represent the across-connection relationship of D-FC strength (left) and D-FC variability (right) versus SC strength, confined to the regional pairs with direct SC. Relevant statistical analyses were performed for all participants based on the SC backbone network. (E) Structural hub probability map for all participants (left) and across-node relationship with the temporal variability of functional degree centrality (right). The hub map was generated by counting the occurrence probability as structural hubs across participants for each region. Notably, brain network analyses here were performed in Session 1 based on AAL-90 atlas, and individual D-FC networks were obtained using a sliding window approach with a window length = 50 s followed by a Bonferroni correction ($p_{corr} < 0.01$). SC: structural connectivity; S-FC: static functional connectivity; D-FC, dynamic functional connectivity; AAL-90, automated anatomical labeling atlas.

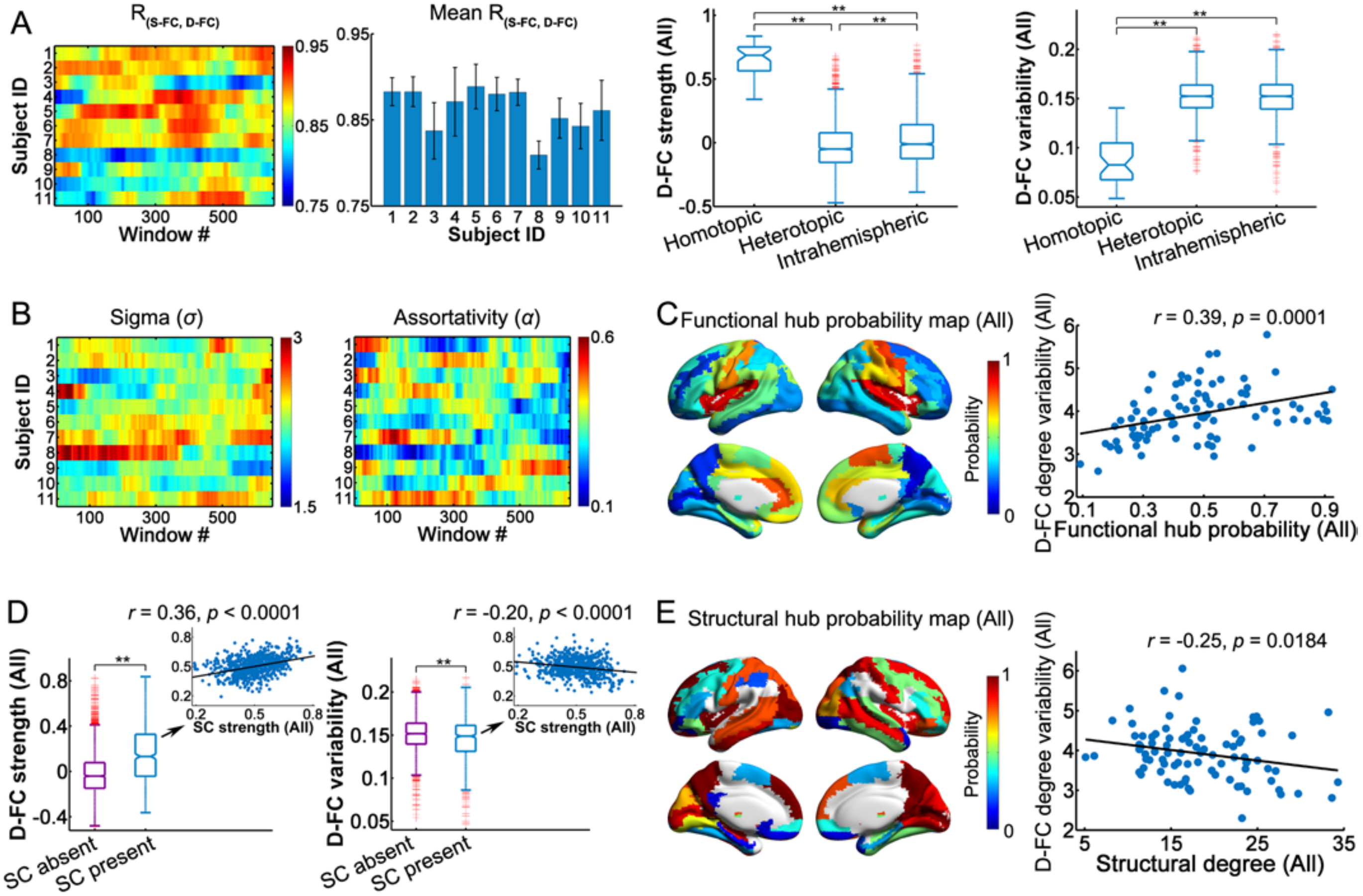

Figure S3. Dynamic characteristics of D-FC brain networks with a window length of 150 s and their structural associations. (A) Spatial similarity between the S-FC and the D-FC matrices over time (left) and the spatial-dependence of D-FC strength and variability for all participants (right). ** Bonferroni corrected $p < 0.001$, 10,000 permutations. (B) Time-varying small-worldness and assortativity of D-FC networks. (C) Functional hub probability map for all participants (left) and scatter plot showing across-node relationship between functional hub probability and the temporal variability of functional degree centrality in the D-FC networks (right). (D) Differences in D-FC strength (left) and temporal variability (right) between region pairs with or without direct SC. ** $p < 0.0001$, 10,000 permutations. Scatter plots (insets) represent the across-connection relationship of D-FC strength (left) and D-FC variability (right) versus SC strength, confined to the regional pairs with direct SC. Relevant statistical analyses were performed for all participants based on the SC backbone network. (E) Structural hub probability map for all participants (left) and across-node relationship with the temporal variability of functional degree centrality (right). The hub map was generated by counting the occurrence probability as structural hubs across participants for each region. Notably, brain network analyses here were performed in Session 1 based on AAL-90 atlas, and individual D-FC networks were obtained using a sliding window approach with a window length = 150 s followed by a Bonferroni correction ($p_{corr} < 0.01$). SC: structural connectivity; S-FC: static functional connectivity; D-FC, dynamic functional connectivity; AAL-90, automated anatomical labeling atlas.

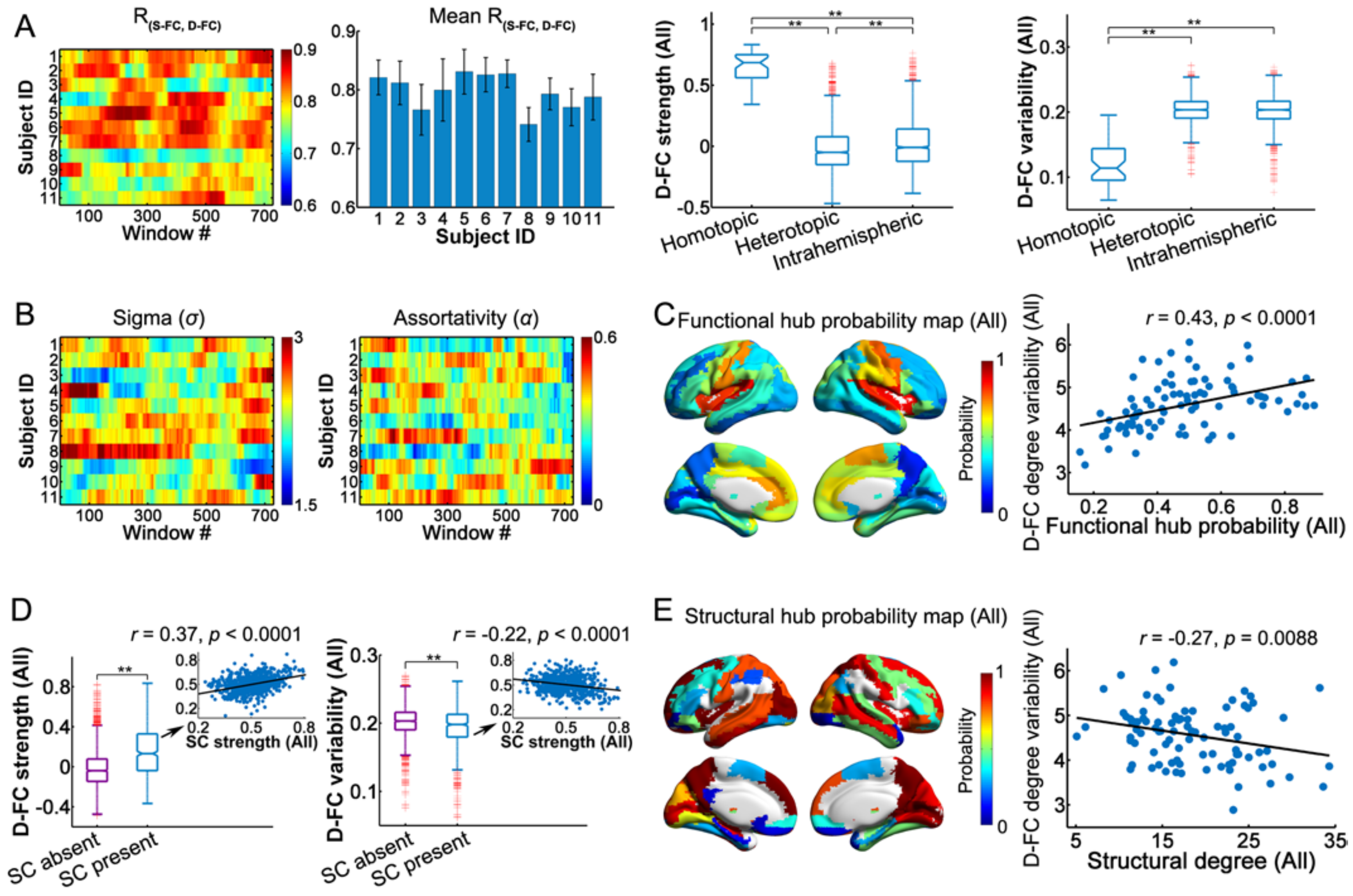

Figure S4. Dynamic characteristics of D-FC brain networks (Bonferroni corrected $p < 0.05$) and their structural associations. (A) Spatial similarity between the S-FC and the D-FC matrices over time (left) and spatial-dependence of D-FC strength and variability for all participants (right). ** Bonferroni corrected $p < 0.001$, 10,000 permutations. (B) Time-varying small-worldness and assortativity of D-FC networks. (C) Functional hub probability map for all participants (left) and scatter plot showing across-node relationship between functional hub probability and the temporal variability of functional degree centrality in the D-FC networks (right). (D) Differences in D-FC strength (left) and temporal variability (right) between region pairs with or without direct SC. ** $p < 0.0001$, 10,000 permutations. Scatter plots (insets) represent the across-connection relationship of D-FC strength (left) and D-FC variability (right) versus SC strength, confined to the regional pairs with direct SC. Relevant statistical analyses were performed for all participants based on the SC backbone network. (E) Structural hub probability map for all participants (left) and across-node relationship with the temporal variability of functional degree centrality (right). The hub map was generated by counting the occurrence probability as structural hubs across participants for each region. Notably, brain network analyses here were performed in Session 1 based on AAL-90 atlas, and individual D-FC networks were obtained using a sliding window approach with a window length = 100 s followed by a Bonferroni correction ($p_{corr} < 0.05$). SC: structural connectivity; S-FC: static functional connectivity; D-FC, dynamic functional connectivity; AAL-90, automated anatomical labeling atlas.

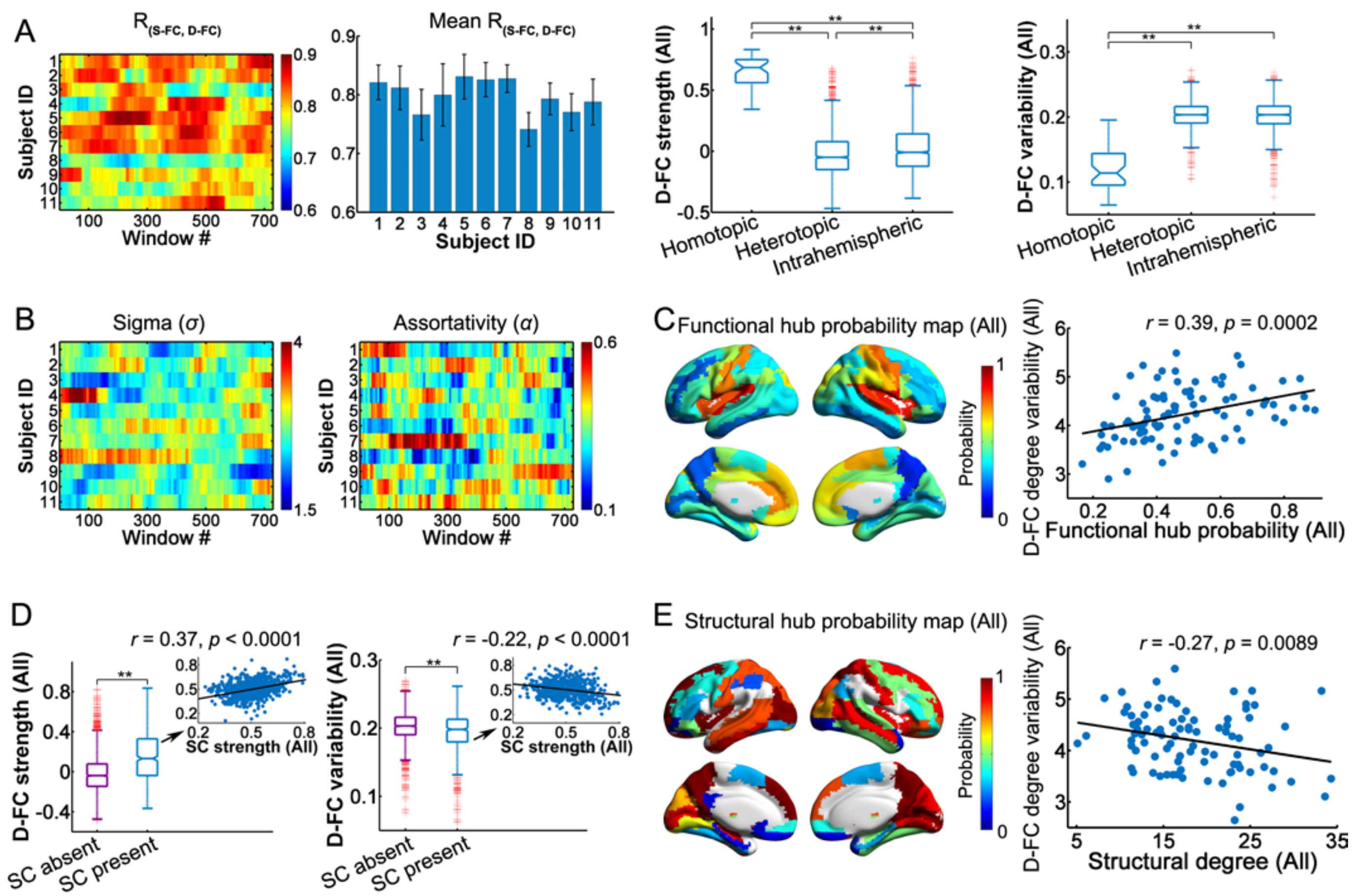

Figure S5. Dynamic characteristics of D-FC brain networks (Bonferroni corrected $p < 0.001$) and their structural associations. (A) Spatial similarity between the S-FC and the D-FC matrices over time (left) and spatial-dependence of D-FC strength and variability for all participants (right). ** Bonferroni corrected $p < 0.001$, 10,000 permutations. (B) Time-varying small-worldness and assortativity of D-FC networks. (C) Functional hub probability map for all participants (left) and scatter plot showing across-node relationship between functional hub probability and the temporal variability of functional degree centrality in the D-FC networks (right). (D) Differences in D-FC strength (left) and temporal variability (right) between region pairs with or without direct SC. ** $p < 0.0001$, 10,000 permutations. Scatter plots (insets) represent the across-connection relationship of D-FC strength (left) and D-FC variability (right) versus SC strength, confined to the regional pairs with direct SC. Relevant statistical analyses were performed for all participants based on the SC backbone network. (E) Structural hub probability map for all participants (left) and across-node relationship with the temporal variability of functional degree centrality (right). The hub map was generated by counting the occurrence probability as structural hubs across participants for each region. Notably, brain network analyses were performed in Session 1 based on AAL-90 atlas, and individual D-FC networks were obtained using a sliding window approach with a window length = 100 s followed by a Bonferroni correction ($p_{corr} < 0.001$). SC: structural connectivity; S-FC: static functional connectivity; D-FC, dynamic functional connectivity; AAL-90, automated anatomical labeling atlas.

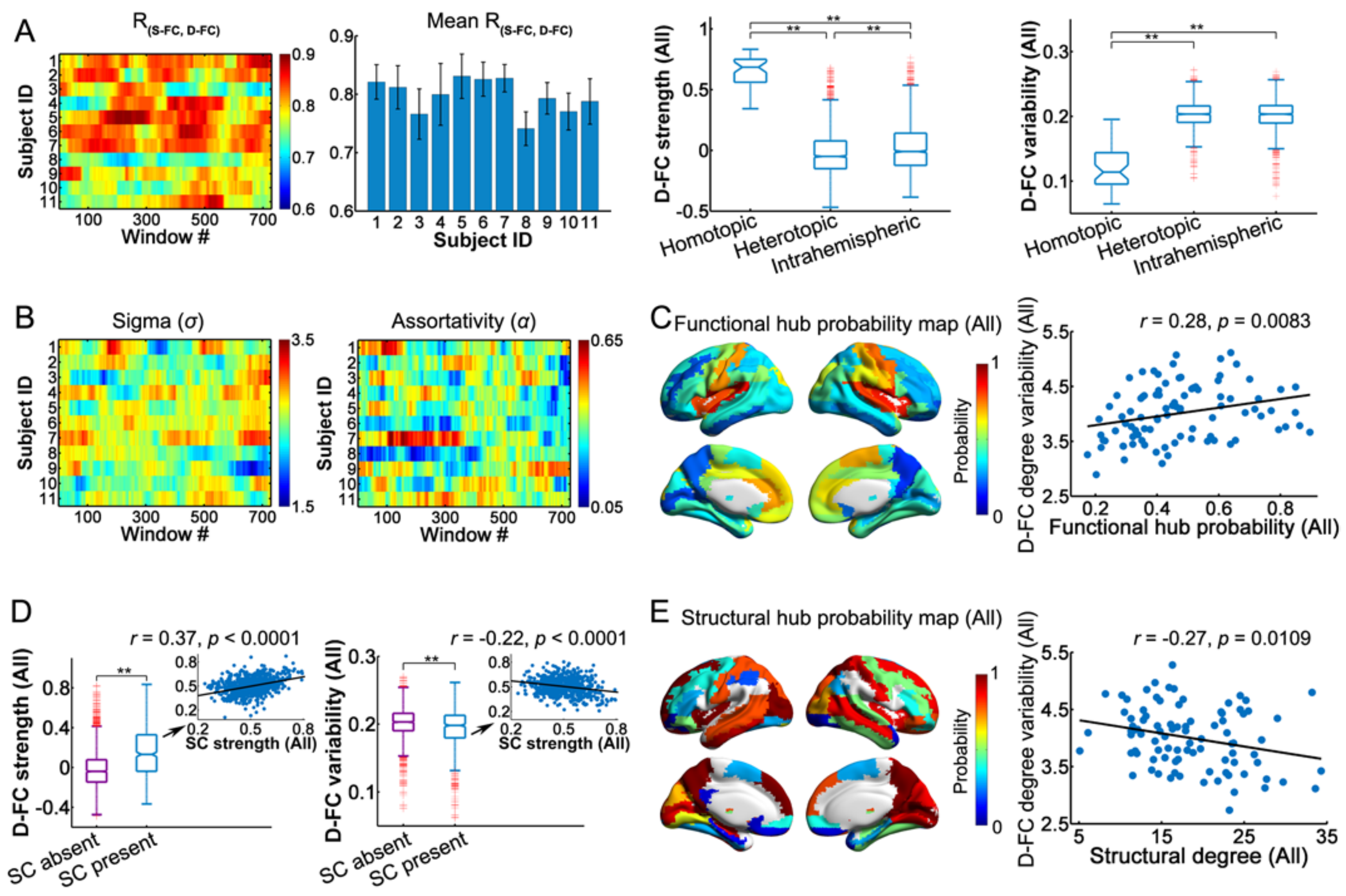

Figure S6. Dynamic characteristics of D-FC brain networks with a fixed sparsity and their structural associations. (A) Spatial similarity between the S-FC and the D-FC matrices over time (left) and spatial-dependence of D-FC strength and variability for all participants (right). ** Bonferroni corrected $p <$ 0.001, 10,000 permutations. (B) Time-varying small-worldness and assortativity of D-FC networks. (C) Functional hub probability map for all participants (left) and scatter plot showing across-node relationship between functional hub probability and the temporal variability of functional degree centrality in the D-FC networks (right). (D) Differences in D-FC strength (left) and temporal variability (right) between region pairs with or without direct SC. ** $p < 0.0001$, 10,000 permutations. Scatter plots (insets) represent the across-connection relationship of D-FC strength (left) and D-FC variability (right) versus SC strength, confined to the regional pairs with direct SC. Relevant statistical analyses were performed for all participants based on the SC backbone network. (E) Structural hub probability map for all participants (left) and across-node relationship with the temporal variability of functional degree centrality (right). The hub map was generated by counting the occurrence probability as structural hubs across participants for each region. Notably, brain network analyses here were performed in Session 1 based on AAL-90 atlas, and individual D-FC networks with a fixed network sparsity were obtained using a sliding window approach with a window length = 100 s. Because similar results were observed under different network sparsity values (10%, 15% and 20%), only results for sparsity = 15% are illustrated here. SC: structural connectivity; S-FC: static functional connectivity; D-FC, dynamic functional connectivity; AAL-90, automated anatomical labeling atlas.

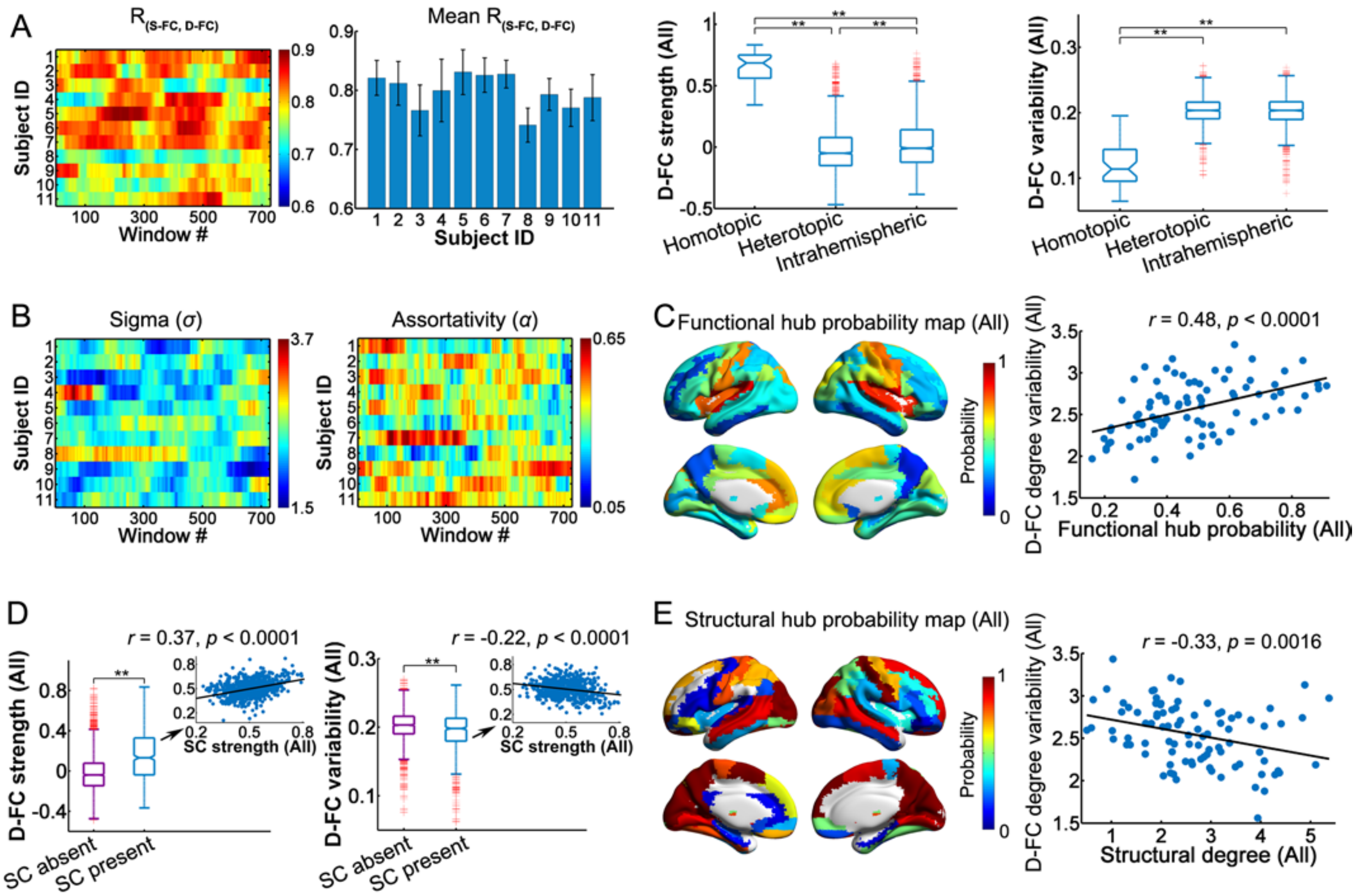

Figure S7. Dynamic characteristics of weighted D-FC brain networks and their structural associations. (A) Spatial similarity between the S-FC and the D-FC matrices over time (left) and the spatial-dependence of D-FC strength and variability for all participants (right). ** Bonferroni corrected $p < 0.001$, 10,000 permutations. (B) Time-varying small-worldness and assortativity of D-FC networks. (C) Functional hub probability map for all participants (left) and scatter plot showing across-node relationship between functional hub probability and the temporal variability of functional degree centrality in the D-FC networks (right). (D) Differences in D-FC strength (left) and temporal variability (right) between region pairs with or without direct SC. ** $p < 0.0001$, 10,000 permutations. Scatter plots (insets) represent the across-connection relationship of D-FC strength (left) and D-FC variability (right) versus SC strength, confined to the regional pairs with direct SC. Relevant statistical analyses were performed for all participants based on the SC backbone network. (E) Structural hub probability map for all participants (left) and across-node relationship with the temporal variability of functional degree centrality (right). For each participant, the structural hubs were identified in weighted structural networks, and then the hub map was generated by counting the occurrence probability as structural hubs across participants for each region. Notably, weighted brain network analyses here were performed in Session 1 based on AAL-90 atlas, and individual weighted D-FC networks were obtained using a sliding window approach with a window length = 100 s followed by a Bonferroni correction ($p_{corr} < 0.01$), meanwhile retaining the correlation coefficients for significant connections. SC: structural connectivity; S-FC: static functional connectivity; D-FC, dynamic functional connectivity; AAL-90, automated anatomical labeling atlas.

Video Legends

Video S1. Time-varying whole-brain functional connectivity patterns across 729 windows for a representative participant (ID: 9). The dynamic functional connectivities among 90 ROIs were obtained using a sliding window correlation approach based on the automated anatomical labeling atlas (Tzourio-Mazoyer et al., 2002). For better visualization of the intra- and inter-hemispheric connectivities, ROIs in left and right hemispheres are reindexed separately, with regions in left hemisphere ranging from 1-45 and those in right hemisphere from 46-90.

Video S2. Time-varying nodal degree centrality across 729 windows for a representative participant (ID: 9). The binary dynamic functional networks of 90 nodes were obtained using a sliding window approach based on the automated anatomical labeling atlas (Tzourio-Mazoyer et al., 2002). The surface-based visualizations of voxel-wise degree maps were made using BrainNet Viewer (http://www.nitrc.org/projects/bnv/, Xia et al. 2013), same hereinafter.

Video S3. Time-varying whole-brain functional connectivity patterns across 729 windows for a representative participant (ID: 9). The dynamic functional connectivities among 1,024 ROIs were obtained using a sliding window correlation approach based on a random parcellation scheme (Zalesky et al., 2010). For better visualization, ROIs were reordered according to their static modular affiliations.

Video S4. Time-varying nodal degree centrality across 729 windows for a representative participant (ID: 9). The binary dynamic functional networks of 1,024 nodes were obtained using a sliding window approach based on a random parcellation scheme (Zalesky et al., 2010).
